# Eigenspace-based reinforcement learning acceleration of statistical convergence in turbulent flows

**DOI:** 10.1016/j.mlwa.2026.100996

**Published:** 2026-09

**Authors:** Núria Masclans, Lluís Jofre

**Affiliations:** Department of Fluid Mechanics, Universitat Politècnica de Catalunya ⋅BarcelonaTech (UPC), Barcelona 08019, Spain

**Keywords:** Scale-resolving flow simulations, Statistics convergence, Turbulence, Reinforcement learning

## Abstract

Direct numerical simulation approaches play a critical role in advancing the understanding and accurate modeling of turbulent flow phenomena. However, the direct simulation of high-Reynolds-number turbulent flows is computationally intensive, requiring high spatial resolution and extended simulation times to achieve statistically reliable results. The strategy leverages deep reinforcement learning to apply dynamic, data-driven perturbations to the Reynolds stresses eigenspace. This allows an optimized control policy to guide the evolution of the Reynolds stresses tensor across all degrees of freedom (magnitude, anisotropy and orientation) within a physics-constrained framework. For the present numerical experiments, a reduced three-dimensional action space targeting magnitude and shape perturbations is utilized as an initial proof-of-concept. To that end, the framework is thoroughly described and its performance evaluated in the context of canonical turbulent channel flows at friction Reynolds numbers $Re_{\tau }=100$ and $Re_{\tau }=180$. The trained agent successfully accelerates the convergence of key flow statistics, including the fluctuating velocity components and the underlying turbulent structure, i.e., anisotropy, particularly in the $Re_{\tau }=100$ training case. A comprehensive computational cost analysis demonstrates that the training overhead is rapidly amortized during unsupervised evaluation phases.Depending on the training duration and early-stopping criteria, the initial training overhead is recovered within 3 to 10 deployment runs under the executed conservative time steps, and within 2 to 5 runs under the projected CFL-optimal conditions. This yields a net computational saving when accelerating the convergence of the fluctuating velocity components to their targeted error tolerances when extrapolating from the $Re_{\tau }=100$ training case to the $Re_{\tau }=180$ deployment case. Furthermore, the evaluation provided critical insights into the complex nature of the control problem, identifying main performance trade-offs during training, the strong dependency on the agent’s actuation frequency, and the fundamental challenges of policy generalization to a new flow regime. This work validates, therefore, the presented approach as a viable tool for targeting the convergence acceleration of specific flow statistics, and demonstrates a clear path toward more computationally efficient high-fidelity simulations of turbulence.

## Highlights

• Novel framework perturbs the Reynolds stress tensor via deep reinforcement learning.
• Reduces high cost of turbulent flow simulations by accelerating statistical convergence.
• Agents guide Reynolds stress tensor properties while satisfying realizability.
• Speeds mean and fluctuating velocity convergence for channel flow at $Re_{\tau }=100$.
• Analyses turbulent structure convergence and generalization capabilities at $Re_{\tau }=180$.

## Graphical abstract

**Figure:**
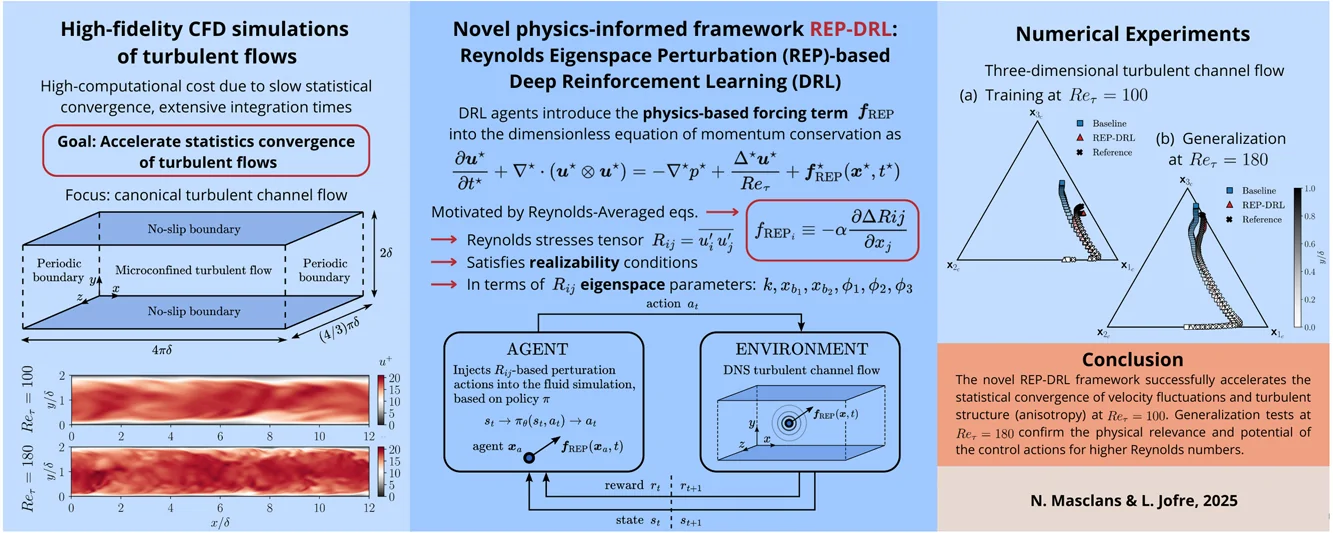

## 1 Introduction

Direct numerical simulation (DNS) is an essential tool in turbulent research, providing detailed high-fidelity data by directly solving the equations of fluid motion without relying on turbulence models. By capturing the full range of spatial and temporal scales, DNS serves as an invaluable resource for validating theoretical predictions and complementing experimental data, especially in regions like near-wall flows where measurements are notoriously difficult (Schultz & Flack, 2013). DNS has been instrumental in studying idealized flows that are challenging to recreate experimentally, offering unparalleled insights into turbulence at high Reynolds numbers, as demonstrated in recent studies with friction Reynolds numbers $Re_{\tau }≳4000$ (Bernardini et al., 2014, Lee and Moser, 2015, Lozano-Durán and Jiménez, 2014). Additionally, these simulations provide a reference for assessing experimental measurement accuracy, and serve as benchmarks for validating cost-effective methods such as large-eddy simulation (LES) and Reynolds-averaged Navier–Stokes (RANS) approaches, together with the corresponding subfilter closures and Reynolds stress models (Pope, 2000). However, the main drawback of DNS is the high computational cost required to obtain statistically reliable (i.e., converged) results. To correctly capture the multi-scale, nonlinear dynamics of turbulent flow, the simulation must resolve all relevant scales of motion, leading to computational demands that scale exponentially with the Reynolds number, e.g., as $Re_{\tau }^{4}$ for wall-bounded flows (Yang et al., 2024). This burden is exacerbated by the long integration times needed for statistical convergence, which can be on the order of $O\left(10^{4}\right)$ large-eddy turnover times (Shirian et al., 2023). The resulting computational, temporal and energy-consumption costs often exceed feasible limits, restricting the practical application of DNS in engineering design contexts, where less accurate but more computational efficient methods are typically preferred.

To address these challenges, novel strategies are required to reduce the computational cost of high-fidelity turbulent flow simulations. In this regard, deep learning has shown considerable potential in accelerating numerical simulations through a variety of techniques, such as speeding up Poisson equation solvers (Chikitkin & Noskov, 2020), reducing domain complexity (Fukami et al., 2019), enhancing the accuracy of finite-difference/volume discretization schemes (Bar-Sinai et al., 2019), implementing time parallelization (Wang et al., 2013), fitting closures to classical turbulence models (Beck et al., 2019), directly replacing the equations of fluid motion with fully-driven machine learning (ML) schemes through deep neural network approximations (Lusch et al., 2018), and using hybrid approaches that integrate ML algorithms within conventional numerical methods to replace or accelerate iterative solvers (Obiols-Sales et al., 2020) and to correct errors in under-resolved simulations (Sirignano et al., 2020). Despite these recent advances, existing methods focus primarily on accelerating the solver’s execution time per step. Consequently, these methods still require extensive integration times to achieve statistical convergence, and often must be tailored to each specific problem. Therefore, to the best of the authors’ knowledge, no general method has yet proven effective in reducing the intrinsic simulation time required to obtain statistically converged data across various applications.

To this end, this work introduces a novel application of the physically constrained eigenspace perturbation framework (EPF) to directly address this gap. Originally developed for uncertainty quantification (UQ) in turbulence modeling (Emory & Iaccarino, 2013), the EPF perturbs the Reynolds stress tensor’s eigenspace, i.e., its eigenvalues and eigenvectors, within realizable physical limits. While primarily used in RANS and LES for UQ (Gorlé and Iaccarino, 2013, Jofre et al., 2018, Jofre et al., 2019), the EPF’s ability to generate a spectrum of physically realizable turbulent states is leveraged here to introduce controlled and distributed perturbations that actively steer the flow toward a statistically converged state. This approach is formulated as an active flow control (ACF) problem, where the control action is introduced into the turbulent flow system as eigenspace perturbations. These flow perturbation actions are constrained only by the realizability conditions of the eigenspace formulation, as no analytical relationship exists between the eigenspace perturbations and the evolution of turbulence statistics. Therefore, the use of deep reinforcement learning (DRL) is particularly well-suited for discovering the optimal actuation strategy, specifically for determining the eigenspace perturbations required to achieve faster statistics convergence in dynamic fluid systems.

The synergy between DRL and ACF has been successfully demonstrated in various experimental and computational flow control problems (Garnier et al., 2021), most commonly implemented in the form of synthetic jet actuators (SJA), which introduce zero net mass flux into the system. This approach was first proposed by (Rabault et al., 2019) for achieving drag reduction for a two-dimensional cylinder at Re=100, and later extended for multi-environment strategies to achieve DRL training parallelization by Rabault and Kuhnle (Rabault & Kuhnle, 2019). By leveraging data collection parallelization, DRL implementations were expanded to tackle more computationally intensive AFC problems, including drag reduction at high Reynolds numbers (Tang et al., 2020, Varela et al., 2022) and multi-agent configurations (Montalà et al., 2024, Suárez et al., 2024). Other studies have applied the combined capabilities of DRL and AFC to turbulent separation bubble control (Font et al., 2024) and two-dimensional Rayleigh-Bénard convection control (Vignon et al., 2023) in canonical problems at low Reynolds numbers.

The key innovation of this work, thus, is the integration of DRL with an AFC strategy based on the EPF to accelerate statistical convergence in turbulent flows. The novelty of this approach lies on its problem formulation: viz. unlike existing approaches that enhance simulation efficiency through solver-specific modifications (such as domain reduction, numerical acceleration, space- and time-parallelization, closures fitting, or modeling), this strategy operates independently of such solver-specific modifications. Instead, it reduces the simulation time required to reach a given convergence tolerance by introducing eigenspace perturbations as an additional, DRL-driven source term in the equations of fluid motion. This Reynolds stresses ($R_{ij}$) eigenspace perturbation (REP)-based deep reinforcement learning (DRL) framework, termed REP-DRL, was initially explored by Masclans and Jofre (2024) for a canonical one-dimensional turbulence (ODT) channel flow at $Re_{\tau }=180$, achieving a 40% reduction in convergence time for the mean streamwise velocity within a supervised RL framework. The current work formally presents and extends the REP-DRL methodology to a high-fidelity, three-dimensional DNS of a turbulent channel flow at $Re_{\tau }=100$ and 180 within an unsupervised DRL setup.

The remainder of the article is structured as follows. Section 2 details the computational framework, presenting the equations of fluid motion and the numerical method employed. Next, Section 3 introduces the Reynolds stresses eigenspace perturbation framework, detailing the theory and application of physically-realizable forcing. Following this, Section 4 formulates the control problem as a Markov decision process (MDP) and describes the complete REP-DRL architecture, including the physical environment, multi-agent design, selected control signals and proximal policy optimization (PPO) algorithm. Section 5, then, presents and discusses the numerical results, including the supervised training analysis, the unsupervised performance and generalization evaluation, a sensitivity analysis of the actuation period, and a comprehensive computational cost and amortization analysis. Finally, Section 6 summarizes the key findings of this work and provides concluding remarks and directions for future work.

## 2 Flow physics modeling

The equations of fluid motion and numerical methods utilized for the direct numerical simulation of turbulent flows are described below.

### 2.1 Equations of fluid motion

The wall-bounded turbulent motion of incompressible fluids is described by the conservation of mass and momentum, which in dimensionless form are written as

(1) $$\nabla^{⋆}\cdot u^{⋆}=0,$$

(2) $$\frac{\partial u^{⋆}}{\partial t^{⋆}}+\nabla^{⋆}\cdot \left(u^{⋆}⊗u^{⋆}\right)=-\nabla^{⋆}p^{⋆}+\frac{\Delta^{⋆}u^{⋆}}{Re_{\tau }},$$

where superscript ⋆ denotes normalized quantities, u is the velocity vector, t is the time, and p is the hydrodynamic pressure. The derivation of these dimensionless equations is based on the following set of inertial-based scalings (Jofre et al., 2020)

(3) $$x^{⋆}=\frac{x}{\delta },\;u^{⋆}=\frac{u}{u_{\tau }},\;t^{⋆}=\frac{u_{\tau }}{\delta }\;t,\;p^{⋆}=\frac{p}{\rho u_{\tau }^{2}},$$

with x the position vector, δ the half-channel height, $u_{\tau }$ the wall friction velocity, and ρ the fluid density. The resulting set of scaled equations includes the friction Reynolds number $Re_{\tau }=\rho u_{\tau }\delta /\mu$, where μ is the dynamic viscosity, which characterizes the ratio between inertial and viscous forces.

### 2.2 Numerical method

The equations of fluid motion introduced in the previous subsection are numerically solved utilizing the in-house flow solver RHEA (Abdellatif et al., 2025, Jofre et al., 2023, Monteiro et al., 2026) by adopting a standard semi-discretization procedure, which involves first discretizing the equations in space and then integrating the resulting system in time. In particular, spatial operators are discretized using second-order central-differencing schemes, and time-advancement is performed by means of a third-order strong-stability preserving (SSP) Runge–Kutta explicit approach (Gottlieb et al., 2001). The convective terms are expanded according to the Kennedy-Gruber-Pirozzoli (KGP) splitting (Coppola, Capuano, and de Luca, 2019, Coppola, Capuano, Pirozzoli, and de Luca, 2019). The method preserves kinetic energy by convection and is locally conservative for mass, momentum, and kinetic energy. This numerical framework provides stable computations without the need for any form of artificial dissipation or stabilization procedures.

## 3 Reynolds stresses eigenspace perturbation framework

This section details the Reynolds stress tensor ($R_{ij}$) eigenspace perturbation framework, which has been developed to be suitable for application within DNS solvers for turbulent flow problems. The framework introduces perturbations locally and instantaneously, ensuring its parallel implementation is efficient and scalable for both structured and unstructured three-dimensional meshes. The methodology can be summarized in five steps: (i) at every DNS time step, the running time-averaged estimate of the Reynolds stress tensor $R_{ij}\left(x,t\right)$ is updated through the corresponding running estimates of the velocity mean and fluctuating components at current averaging time; (ii)–(v) at every discrete control time instance $t_{m}$, the following operations are performed based on the most recent estimate of $R_{ij}$: (ii) $R_{ij}$ is decomposed into its magnitude, shape, and orientation; (iii) these components are parameterized using physically meaningful variables, i.e., turbulent kinetic energy, barycentric coordinates, and eigenvectors Euler angles; (iv) perturbations are introduced in this parametric space, maintaining physical realizability; and (v) the perturbed $R_{ij}$ is reconstructed and used to derive the instantaneous forcing term, which is introduced into the equations of fluid motion to manipulate the flow dynamics. By relying exclusively on the local, intrinsic properties of the stress tensor eigenspace at each spatial point x, the mathematical core of this framework remains entirely decoupled from any global geometric constraints or turbulent channel flow simplifications. As mentioned in Section 1, the foundation of this physics-based approach lies in the pioneering work of (Emory and Iaccarino, 2013, Emory et al., 2011, Gorlé and Iaccarino, 2013, Jofre et al., 2018, Jofre et al., 2019) for the UQ of turbulence models.

### 3.1 Reynolds stresses realizability conditions

In the direct numerical simulation of turbulent flows, the instantaneous velocity u is described by Eqs. (1), (2). However, the target quantity for this framework is the Reynolds stress tensor $R_{ij}$, a second-order statistical moment derived from time-averaging the equations of fluid motion.

The Reynolds stress tensor is formally defined based on the Reynolds decomposition $u\left(x,t\right)=\bar{u}\left(x\right)+u^{\prime }\left(x,t\right)$, with $\bar{\left(\cdot \right)}$ denoting the temporal averaging operator, which yields

(4) $$R_{ij}\left(x\right)=\bar{u_{i}^{\prime }\left(x,t\right)u_{j}^{\prime }\left(x,t\right)},$$

where $\bar{u}\left(x\right)$ is the mean velocity and $u^{\prime }\left(x,t\right)$ represents the velocity fluctuation. The trace of this tensor, $R_{ii}$, is twice the turbulent kinetic energy (TKE), which is defined as $k=\frac{1}{2}R_{ii}=\frac{1}{2}\bar{u_{i}^{\prime }u_{i}^{\prime }}$. Therefore, for $R_{ij}$ to be physically plausible, the tensor must remain symmetric and positive semi-definite (Schumann, 1977), which ensures non-negative turbulent kinetic energies. These requirements are equivalent to the realizability conditions (Speziale et al., 1993)

(5) $$R_{\alpha \alpha }\geq 0\;\text{for}\;\alpha \in \left\{1,2,3\right\},$$

(6) $$R_{\alpha \beta }^{2}\leq R_{\alpha \alpha }R_{\beta \beta }\;\text{for}\;\alpha \neq \beta ,$$

(7) $$\det\left(R_{ij}\right)\geq 0.$$

In this study, $R_{ij}\left(x,t\right)$ is not evaluated as a fully converged long-time average, but rather as a running time-averaged estimate that is updated at every DNS time step. In practice, this running time-averaged estimate is constructed from the running time-averaged estimation of the velocity fluctuations, $u_{i}^{\prime }$, which are themselves dependent on the running estimations of the mean velocity components, ${\bar{u}}_{i}$. Accordingly, $R_{ij}$ is incrementally updated from the products of the running time-averaged estimations for the velocity fluctuations, $u_{i}^{\prime }u_{j}^{\prime }$, ensuring a smoothly temporal evolution of the second-order turbulence statistics as new flow data become available. Finally, although the running time-averaged estimation of $R_{ij}$ is updated at every DNS time step, all derived quantities used in later subsections, including the turbulent kinetic energy k, the anisotropy tensor $a_{ij}$, and the eigenspace variables, are evaluated only at discrete control instances from this more frequently updated running estimate of $R_{ij}$.

### 3.2 Reynolds stresses eigendecomposition

The focus of this work is to introduce optimal perturbations into the converging, time-averaged Reynolds stresses tensor, $R_{ij}$, to achieve statistical convergence within reduced simulation times. To ensure the perturbation framework is physically interpretable, $R_{ij}$ is decomposed into six components that represent its amplitude (trace), shape (eigenvalues), and orientation (eigenvectors). This procedure begins with the eigendecomposition of the symmetric and trace-free normalized anisotropy tensor, $a_{ij}$, defined as

(8) $$a_{ij}=\left(\frac{R_{ij}}{R_{kk}}-\frac{1}{3}\delta_{ij}\right)=v_{in}\Lambda_{nl}v_{jl},$$

where $R_{kk}$ is the trace of $R_{ij}$, $v_{in}$ is the matrix of orthonormal eigenvectors $\left(v_{1},v_{2},v_{3}\right)$, and $\Lambda_{nl}$ is the traceless diagonal matrix of eigenvalues $\left(\lambda_{1},\lambda_{2},\lambda_{3}\right)$. Note that the eigenvalues satisfy the zero-sum condition due to the trace-free property of $a_{ij}$. Additionally, the eigenvalues and their corresponding eigenvectors are ordered such that $\lambda_{1}\geq \lambda_{2}\geq \lambda_{3}$. Enforcing the realizability conditions [Eqs. (5)–(7)] on this decomposition imposes the following bounds on the components of $a_{ij}$ (Jofre et al., 2018, Jofre et al., 2019)

(9) $$-\frac{1}{3}\leq a_{\alpha \alpha }\leq \frac{2}{3}\;\text{for}\;\alpha \in \left\{1,2,3\right\},$$

(10) $$-\frac{1}{2}\leq a_{\alpha \beta }\leq \frac{1}{2}\;\text{for}\;\alpha \neq \beta .$$

Finally, the Reynolds stresses tensor, $R_{ij}$, can be reconstructed as

(11) $$R_{ij}=2k\left(v_{in}\;\Lambda_{nl}\;v_{jl}+\frac{1}{3}\delta_{ij}\right),$$

where the amplitude, shape, and orientation of $R_{ij}$ are represented by the turbulent kinetic energy $\left(k=R_{kk}/2\right)$, the anisotropy tensor eigenvalues $\left(\lambda_{l}\right)$, and the eigenvectors $\left(v_{l}\right)$, respectively. This decomposition, together with the zero-sum property of the eigenvalues, allows the representation of $R_{ij}$ in a parametric space of six independent, physically interpretable variables: $k,\lambda_{1},\lambda_{2},v_{1},v_{2}$, and $v_{3}$. To enhance physical interpretability and facilitate the enforcement of realizability, the eigenvalues are represented using barycentric mapping, and the eigenvectors are described through their Euler angles, as detailed in the following subsections.

### 3.3 Barycentric mapping for eigenvalues parameterization

The barycentric map, developed by Banerjee et al. (2007), provides an effective approach to interpret, visualize, and enforce realizability conditions on the anisotropy tensor. This method establishes an affine mapping between the anisotropy eigenvalues, $\lambda =\left(\lambda_{1},\lambda_{2},\lambda_{3}\right)$, and the barycentric coordinates, $x=\left(x_{1},x_{2}\right)$, defined as

(12) $$x=x_{1c}\left(\lambda_{1}-\lambda_{2}\right)+2\;x_{2c}\left(\lambda_{2}-\lambda_{3}\right)+x_{3c}\left(3\lambda_{3}+1\right).$$

This mapping, combined with the zero-sum condition of the eigenvalues, is an invertible and unique transformation compactly expressed as $x_{i}=B_{in}\lambda_{n}+x_{{3c}_{i}}$, where $B\in R^{2\times 3}$ is the transformation matrix and $x_{3c}\in R^{2}$ is the constant offset vector representing the isotropic state. As illustrated in Fig. 1, $x_{1c}=\left(0,0\right)$, $x_{2c}=\left(1,0\right)$, and $x_{3c}=\left(1/2,\sqrt{3}/2\right)$ correspond to the three vertices of the barycentric triangle, each representing a distinct limiting state of turbulence: one-component (rod-like, $x_{1c}$), two-component (disk-like, $x_{2c}$), and three-component (isotropic, $x_{3c}$) turbulence states.

As a result, any realizable state of turbulence can be interpreted as a convex combination of these limiting states, requiring that x lies within the barycentric triangle. This constraint not only ensures compliance with the realizability conditions, but also simplifies their visualization and enforcement.

Fig. 1Barycentric map based on the eigenvalues of a general second-order anisotropy tensor. (a) Limiting states of componentiality. (b) Tensor shapes visualized with superquadric glyphs (Kindlmann, 2004) (figure regenerated using open-source software (Kindlmann et al., 2003)).Fig. 1

**(a):**
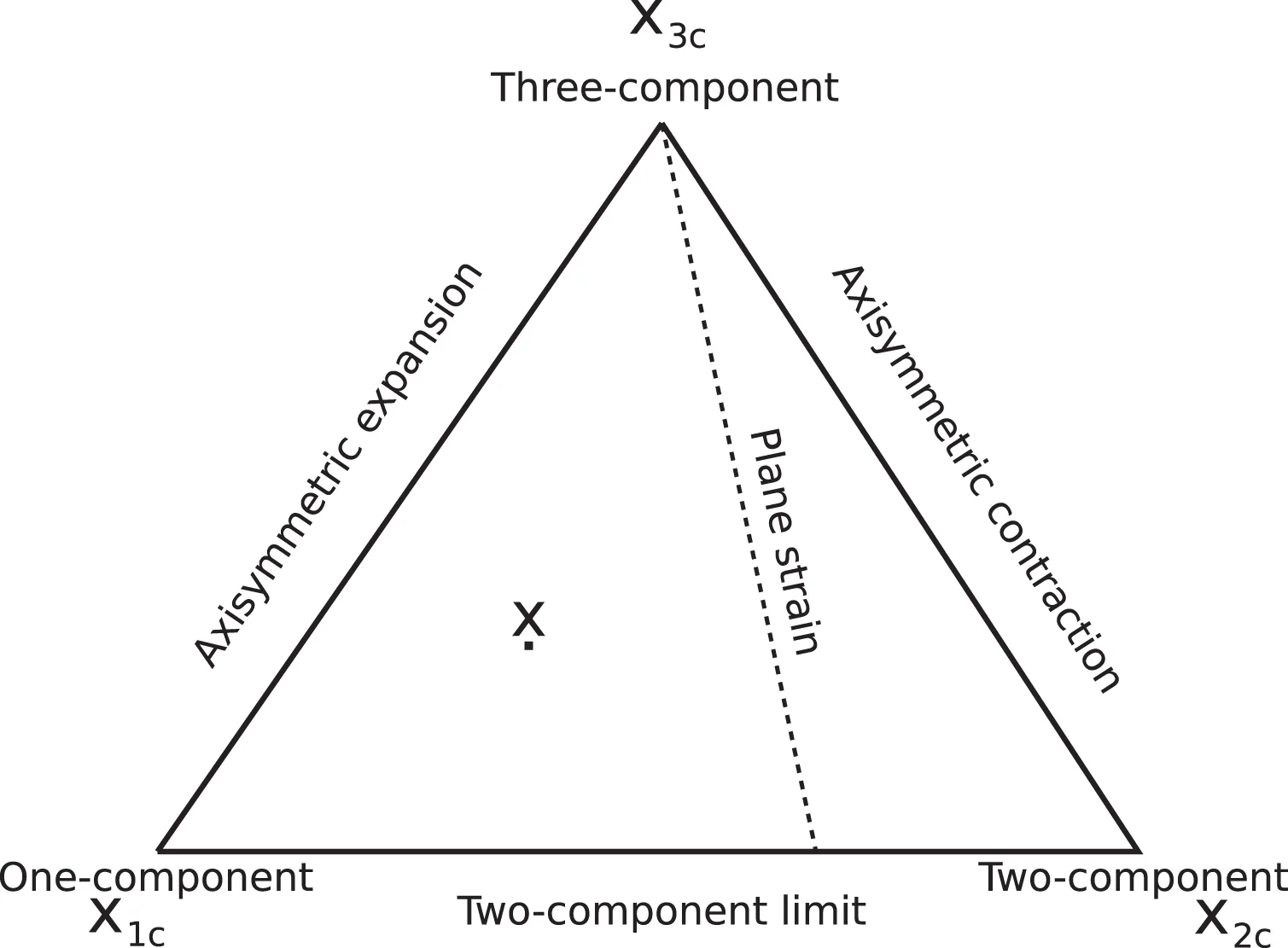
.

**(b):**
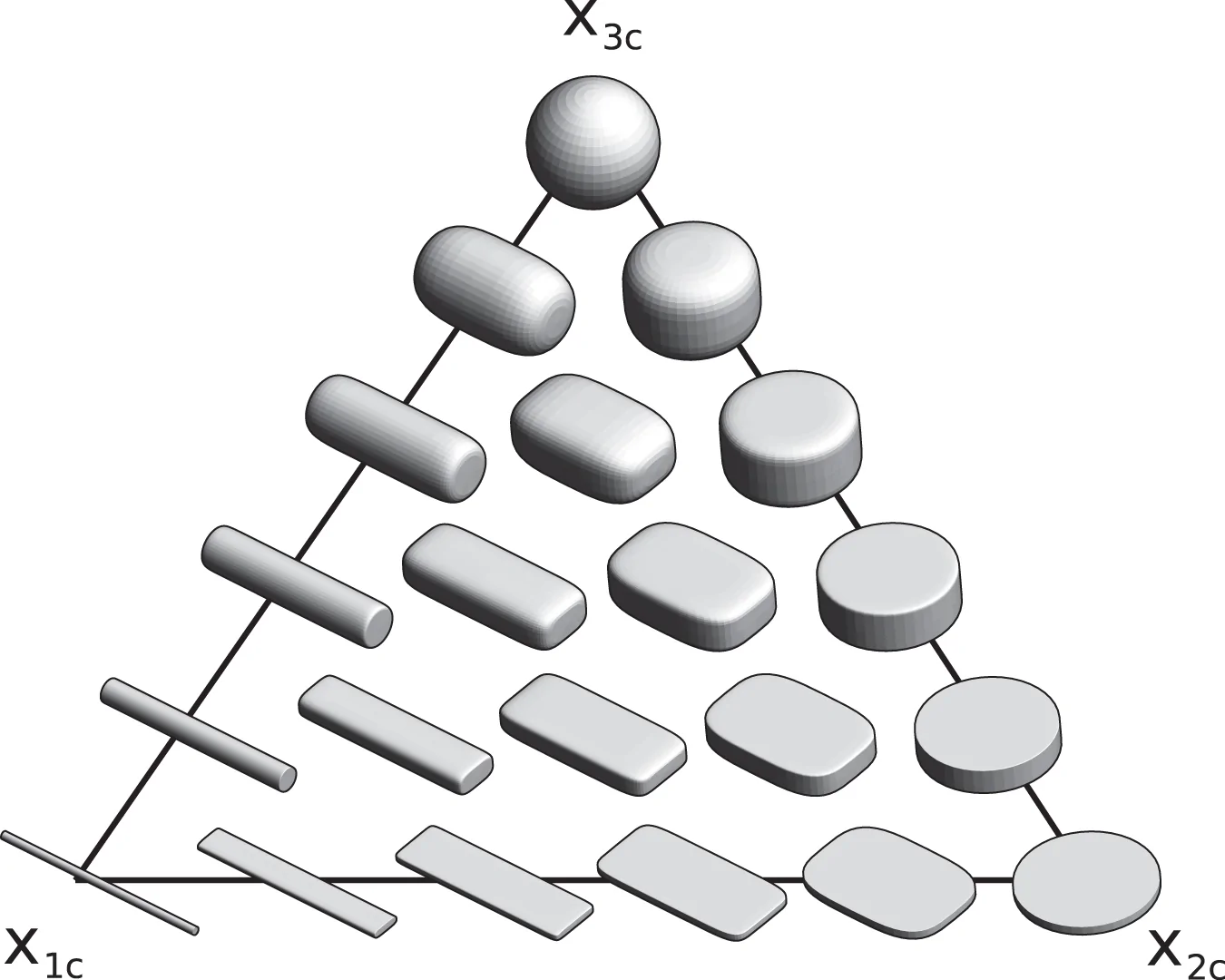
.

### 3.4 Euler rotation angles for eigenvectors parameterization

A parameterization scheme is required to describe and perturb the eigenvector matrix $Q_{ij}$, which defines the orientation of the Reynolds stresses anisotropy tensor, $a_{ij}$. This matrix is constrained by orthogonality and unit-length conditions, thereby reducing the nine elements of $Q_{ij}$ to three independent parameters. As a result, the eigenvectors frame can be treated as a rigid body, whose orientation is fully described by these three degrees of freedom. Following the approach of Xiao et al. (2017) for injecting perturbations into the eigenvectors tensor, the orientation of the eigenvectors frame is parameterized using Euler angles. These angles describe the transformation from the global coordinate system to the local eigenvectors frame through three successive rotations, performed in a specific sequence. In this work, the intrinsic $z-x^{\prime }-z^{\prime \prime }$ convention is adopted, where rotations are performed about the axes of the local coordinate system as it evolves with each rotation. Starting with the eigenvectors frame XYZ initially aligned with the global reference system xyz: (i) the first rotation, by an angle $\phi_{1}$, is performed about the z-axis, resulting in the intermediate local coordinate system $x^{\prime }y^{\prime }z^{\prime }$; (ii) the second rotation, by an angle $\phi_{2}$, is about the $x^{\prime }$-axis, producing the intermediate system $x^{\prime \prime }y^{\prime \prime }z^{\prime \prime }$; and (iii) the third rotation, by an angle $\phi_{3}$, is about the $z^{\prime \prime }$-axis, yielding to the desired eigenvectors frame XYZ. These transformations can be mathematically represented by the product of three rotation matrices as

(13) $$Q=R_{z}\left(\phi_{1}\right)R_{x^{\prime }}\left(\phi_{2}\right)R_{z^{\prime \prime }}\left(\phi_{3}\right),$$

where $R_{z}\left(\phi_{1}\right)$, $R_{x^{\prime }}\left(\phi_{2}\right)$, and $R_{z^{\prime \prime }}\left(\phi_{3}\right)$ are the elemental rotation matrices, whose specific mathematical formulation is detailed in Goldstein (1980). Therefore, the Euler angles $\phi_{1}$, $\phi_{2}$, and $\phi_{3}$ provide a precise and systematic parameterization of the eigenvectors matrix, enabling an efficient framework for analyzing and perturbing the orientation of the Reynolds stresses tensor.

### 3.5 Reynolds stresses perturbation

The eigendecomposition of $R_{ij}$, combined with the parameterization schemes detailed in the previous subsections, enables a systematic representation of the tensor in terms of six physically meaningful scalar fields: the turbulent kinetic energy (k) representing its magnitude, the barycentric coordinates ($x_{1},\;x_{2}$) describing its shape, and the Euler angles ($\phi_{1},\;\phi_{2},\;\phi_{3}$) defining its orientation. These variables collectively define the eigenspace vector, denoted as $q=\left(k,\;x_{1},\;x_{2},\;\phi_{1},\;\phi_{2},\;\phi_{3}\right)$. This physics-based parameterization provides a robust framework for perturbing $R_{ij}$, offering insights into how each component perturbation of $R_{ij}$ contributes to the statistical properties of the flow. In this regard, perturbations are introduced directly into the six-dimensional parametric space by adding a discrepancy vector Δq as

(14) $$\overset{\sim }{q}\left(x,t\right)=q\left(x,t\right)+\Delta q\left(x,t\right),$$

where $\Delta q=\left(\Delta k,\;\Delta x_{1},\;\Delta x_{2},\;\Delta \phi_{1},\;\Delta \phi_{2},\;\Delta \phi_{3}\right)$ represents the eigenspace perturbation vector, and $\overset{\sim }{q}=\left(\overset{\sim }{k},\;\overset{\sim }{x_{1}},\;\overset{\sim }{x_{2}},\;\overset{\sim }{\phi_{1}},\;\overset{\sim }{\phi_{2}},\;\overset{\sim }{\phi_{3}}\right)$ is the resulting perturbed eigenspace vector. Physical realizability is inherently enforced by constraining $\overset{\sim }{q}$: the perturbed turbulent kinetic energy must remain non-negative ($\overset{\sim }{k}\geq 0$), and the perturbed barycentric coordinates $\left({\overset{\sim }{x}}_{1},{\overset{\sim }{x}}_{2}\right)$ must lie within the barycentric triangle.

Notably, the eigendecomposition of $R_{ij}$ and all derived operations required to obtain the perturbed eigenspace vector $\overset{\sim }{q}$ are not performed at every DNS time step, but only at discrete control instances $t_{m}$, separated by a fixed control interval $t_{\text{act}}$ (see Table 2). At each control step, the tensor $R_{ij}$ is obtained from the most recent running time-averaged estimate of the Reynolds stresses, which is continuously updated throughout the simulation. This procedure ensures that the Reynolds stresses eigendecomposition and the subsequent perturbation of the eigenspace variables are consistently applied to the latest available $R_{ij}$ estimate, while clearly separating the fast DNS time scale from the slower reinforcement learning control updates. From this eigenspace representation at each control step, the components of $\overset{\sim }{q}$ are then used to reconstruct the new, perturbed Reynolds stresses tensor ${\overset{\sim }{R}}_{ij}$. Finally, the resulting dimensionless $R_{ij}$ perturbation tensor, $\Delta R_{ij}^{⋆}$, represents the targeted change to the local state of turbulence, and is computed as the difference $\Delta R_{ij}^{⋆}={\overset{\sim }{R}}_{ij}^{⋆}-R_{ij}^{⋆}$. This dimensionless $R_{ij}^{⋆}$ perturbation, $\Delta R_{ij}^{⋆}$, is introduced into the instantaneous flow field by incorporating a $R_{ij}^{⋆}$ eigenspace perturbation (REP)-based forcing term, $f_{\text{REP}}^{⋆}$, into the dimensionless momentum Eq. (2), yielding the forced momentum equation as

(15) $$\frac{\partial u^{⋆}}{\partial t^{⋆}}+\nabla^{⋆}\cdot \left(u^{⋆}⊗u^{⋆}\right)=-\nabla^{⋆}p^{⋆}+\frac{\Delta^{⋆}u^{⋆}}{Re_{\tau }}+f_{\text{REP}}^{⋆}\left(x^{⋆},t^{⋆}\right).$$

The physical basis for the form of $f_{\text{REP}}^{⋆}$ is motivated by the Reynolds-Averaged Navier–Stokes (RANS) equations, where the Reynolds stresses tensor $R_{ij}$ appears explicitly within a divergence term. The inertial-based scaling defined in Eq. (3) are employed, which yields the additional scalings $R_{ij}^{⋆}=R_{ij}/u_{\tau }^{2}$ and $f_{\text{REP}}^{⋆}=\left(\delta /u_{\tau }^{2}\right)f_{\text{REP}}$. Therefore, in this Reynolds-averaged framework, the dimensionless REP-based forcing ($f_{\text{REP}}^{⋆}$) is expressed as the divergence of the Reynolds stresses perturbations ($\Delta R_{ij}^{⋆}$) as

(16) $$\frac{\partial {\bar{u}}_{i}^{⋆}}{\partial t^{⋆}}+{\bar{u}}_{j}^{⋆}\frac{\partial \;{\bar{u}}_{i}^{⋆}}{\partial x_{j}^{⋆}}=\frac{1}{Re_{\tau }}\frac{\partial^{2}\;{\bar{u}}_{i}^{⋆}}{\partial x_{j}^{⋆2}}-\frac{\partial \;{\bar{p}}^{⋆}}{\partial x_{i}^{⋆}}-\frac{\partial R_{ij}^{⋆}}{\partial x_{j}^{⋆}}+{\bar{f}}_{{\text{REP}}_{i}}^{⋆},$$

(17) $${\bar{f}}_{{\text{REP}}_{i}}^{⋆}=-\frac{\partial \Delta R_{ij}^{⋆}}{\partial x_{j}^{⋆}}.$$

Applying this mean-derived forcing [Eq. (17)] directly to the instantaneous field [Eq. (15)] is physically validated by a distinct separation of timescales. In particular, the forcing term $f_{\text{REP}}^{⋆}$ is constructed from slow-evolving running statistics of the local mean and fluctuating velocity components. Although the control policy updates $f_{\text{REP}}^{⋆}$ at discrete time intervals ($\Delta t^{+}=4.5$ to 5, denoted as $t_{\text{act}}^{+}$ in Table 2), the high statistical inertia of the running averages ensures that the state and reward policy inputs, and consequently the resulting policy actions, remain highly correlated between successive control steps. Furthermore, the resulting forcing terms are temporally smoothed between subsequent control steps (as detailed in Section 4.3.3) to ensure a stable, continuous evolution in time. Consequently, the instantaneous forcing $f_{\text{REP}}^{⋆}$ varies smoothly on a macroscopic timescale and lacks any direct correlation with instantaneous fields and high-frequency, stochastic turbulent fluctuations. Therefore, in a standard Reynolds decomposition ($f_{\text{REP}}^{⋆}=\bar{f_{\text{REP}}^{⋆}}+f_{\text{REP}}^{\prime ⋆}$), the fluctuating component of the forcing term is negligible ($f_{\text{REP}}^{\prime ⋆}\approx 0$), yielding a direct correspondence between the applied instantaneous force and its Reynolds average ($f_{\text{REP}}^{⋆}\approx \bar{f_{\text{REP}}^{⋆}}$). Accordingly, the dimensionless, instantaneous forcing vector is defined as

(18) $$f_{{\text{REP}}_{i}}^{⋆}\equiv -\alpha \frac{\partial \Delta R_{ij}^{⋆}}{\partial x_{j}^{⋆}},$$

where α is a non-dimensional relaxation factor that controls the magnitude of the applied forcing; α=0 recovers the original, unperturbed momentum Eq. (2).

This formulation seamlessly integrates the physics-based perturbation framework into the equations of fluid motion. The main challenge, however, is determining the optimal temporal evolution of the perturbation tensor, $\Delta R_{ij}^{⋆}\left(x^{⋆},t^{⋆}\right)$, required to drive the flow statistics to convergence most efficiently. Deriving a closed-form analytical expression for these flow perturbations is infeasible due to the inherently nonlinear, multiscale dynamics of turbulent flows. Furthermore, any viable perturbation policy must ensure that $\partial \Delta R_{ij}^{⋆}/\partial x_{j}^{⋆}$ (and thus $f_{\text{REP}}^{⋆}$) decays to zero as the system approaches the convergence state, so as not to alter the final statistical properties.

While this decay behavior is mathematically essential in long-term integration applications to ensure the perturbation forcing vanishes once the flow statistics reach convergence, the operational time windows in the present study are intentionally kept short ($t^{+}∼10^{2}$, as detailed in Section 5). Under these conditions, the baseline statistics remain far from traditional convergence limits, requiring $f_{\text{REP}}^{⋆}$ to act as an active, continuous driving force to rapidly reshape the flow statistics. Notably, because $f_{\text{REP}}^{⋆}\neq 0$ throughout these active control windows, the accumulated running time-averaged statistics sample the forced flow dynamics rather than the unforced dynamics. While the accelerated statistical gain achieved during control is retained within the cumulative history of the target statistics at the end of the control window, this formulation does not assume that the instantaneous unforced trajectory of the target flow fields remains on this forced path once control is deactivated. Therefore, upon forcing deactivation ($f_{\text{REP}}^{⋆}=0$), the physical flow is expected to undergo transient relaxation back toward the unforced baseline dynamics.

To address this complex, high-dimensional optimization problem, this work employs a reinforcement learning (RL) framework. This framework aims to discover an optimal policy that, at each control step, determines the optimal six-component perturbation vector $\Delta q=\left(\Delta k,\;\Delta x_{1},\;\Delta x_{2},\;\Delta \phi_{1},\;\Delta \phi_{2},\;\Delta \phi_{3}\right)$ for accelerating the flow’s statistics convergence. As detailed in this section, this perturbation vector, Δq, directly defines the realizable perturbation tensor $\Delta R_{ij}^{⋆}$, which in turn defines the forcing term $f_{\text{REP}}^{⋆}$ (18) applied to the instantaneous flow (15).

## 4 Deep reinforcement learning methodology

This section details the complete deep reinforcement learning methodology, from its high-level justification to its specific algorithmic implementation. This section begins by motivating the use of a DRL-based approach, highlighting the limitations of conventional control strategies for this problem. Next, the physical problem environment, the DNS of a turbulent channel flow, is defined. This physical problem is then formulated as a Markov decision process (MDP), with explicit definitions for the agent distribution, state and action spaces, and reward function. By design, the presented multi-agent configuration is highly modular; the localized agent distribution can be readily adapted to alternative flow geometries, while the error-based state and reward signals can be retargeted to different flow statistics. Finally, the specific deep reinforcement learning algorithm chosen to solve this Markov decision process, proximal policy optimization, is introduced and its selection is justified.

### 4.1 Motivation & framework

As established in the previous section, determining the optimal $R_{ij}$ eigenspace perturbation (REP)-based forcing, $f_{\text{REP}}\left(x,t\right)$, to accelerate statistical convergence is a complex optimal control problem for which no analytical solution exists. An effective control policy must meet two primary requirements: (i) adaptive to the flow’s evolving state, and (ii) capable of influencing a broad range of turbulence scales. These requirements, combined with the high-dimensional state space and complex, nonlinear dynamics, render conventional control strategies impractical. Simple, pre-tuned controllers that rely on extensive, problem-specific parameter fine-tuning are computationally prohibitive in such turbulent flow systems, especially at high Reynolds numbers. More advanced strategies also face critical limitations. For instance, model-based control techniques (Moerland et al., 2023), which rely on an explicit, reduced-order model (ROM) of the system dynamics, would require a model capable of accurately predicting the evolution of the turbulence statistics as a function of the forcing term, a significant modeling challenge in itself. Furthermore, approaches based on *offline* supervised learning would require a prohibitively large, pre-computed dataset of control strategies, which is infeasible to generate using computationally expensive DNS (Brunton et al., 2020).

These limitations motivate the use of a data-driven, model-free optimization framework such as deep reinforcement learning. In recent years, DRL has emerged as a powerful tool for complex flow control and optimization problems (Novati et al., 2017, Verma et al., 2018), in some cases demonstrating superior performance to traditional strategies (Novati et al., 2019). The DRL framework is particularly well-suited for this challenge, as it enables the discovery of an optimal control policy through direct, iterative interaction with the high-fidelity simulation. By maximizing a cumulative reward signal, this approach allows the policy to adapt to the flow’s dynamics without an *a priori* model or dataset. The DRL framework proposed in this work is, therefore, designed to solve this optimal control problem. Specifically, the $R_{ij}$ eigenspace perturbation (REP)-based deep reinforcement learning (REP-DRL) framework is introduced, which leverages the $R_{ij}$ eigenspace perturbation methodology detailed in Section 3. The objective is to discover an optimal policy that determines the sequence of eigenspace perturbations Δq(x,t) which in turn generates the forcing term $f_{\text{REP}}\left(x,t\right)$ required to accelerate the convergence of turbulence statistics while preserving the properties of the final converged state. This approach provides a novel method to improve the computational efficiency of high-accuracy flow solvers, applied here to the DNS of a three-dimensional turbulent channel flow detailed in the following subsection.

**Table 1:** Physical and numerical parameters for the DNS of the turbulent channel flow simulations.

Parameter	Case I ($Re_{\tau }=100$)	Case II ($Re_{\tau }=180$)
*Physical Properties*		
Friction Reynolds number, $Re_{\tau }$	100	180
Friction velocity, $u_{\tau }$ (m/s)	1.0	
Channel half-height, δ (m)	1.0	
Density, ρ (kg/m^3^)	1.0	
Dynamic viscosity, μ (kg/m⋅s)	0.01	1/180
Kinematic viscosity, ν=μ/ρ (m^2^/s)	0.01	1/180

*Computational Domain*		
Domain size ($L_{x}\times L_{y}\times L_{z}$)	4πδ×2δ×(4/3)πδ	
Grid resolution ($N_{x}\times N_{y}\times N_{z}$)	64×64×64	256×128×128
Grid type (x,z-directions)	Uniform	Uniform
Grid type (y-direction)	Uniform	Stretched toward walls
Wall-unit resolution ($\Delta x^{+},\Delta y^{+},\Delta z^{+}$)	19.6,3.1,6.5	8.8,(0.2−4),5.9

*Time Stepping*		
Time step, Δt (s)	$10^{-4}$	$5\cdot 10^{-5}$
Viscous time scale, $t_{\tau }$ (s)	0.01	1/180
Viscous time step (conservative), $\Delta t_{c}^{+}$	$10^{-2}$	$9\cdot 10^{-3}$
Viscous time step (CFL=0.5), $\Delta t_{op}^{+}$	$5.4\cdot 10^{-1}$	$2.3\cdot 10^{-1}$

**Fig. 2:**
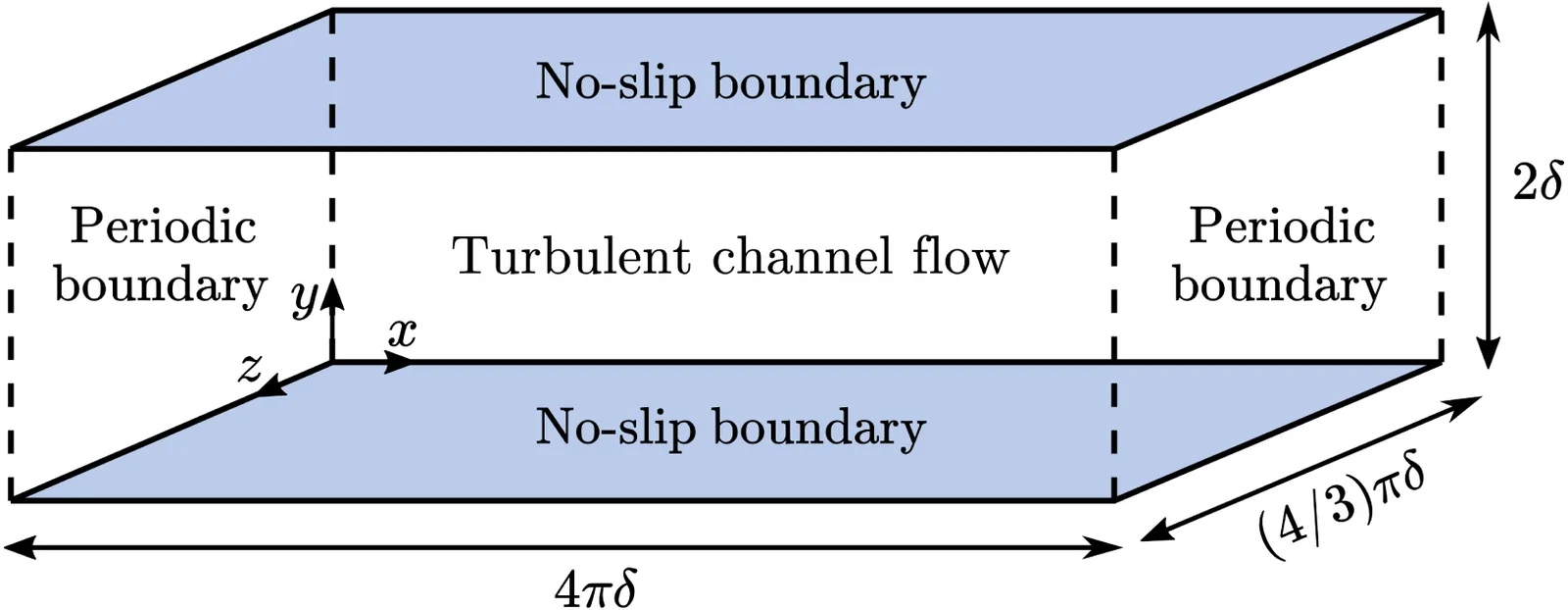
Schematic of the computational domain for the turbulent channel flow setup.

**Fig. 3:**
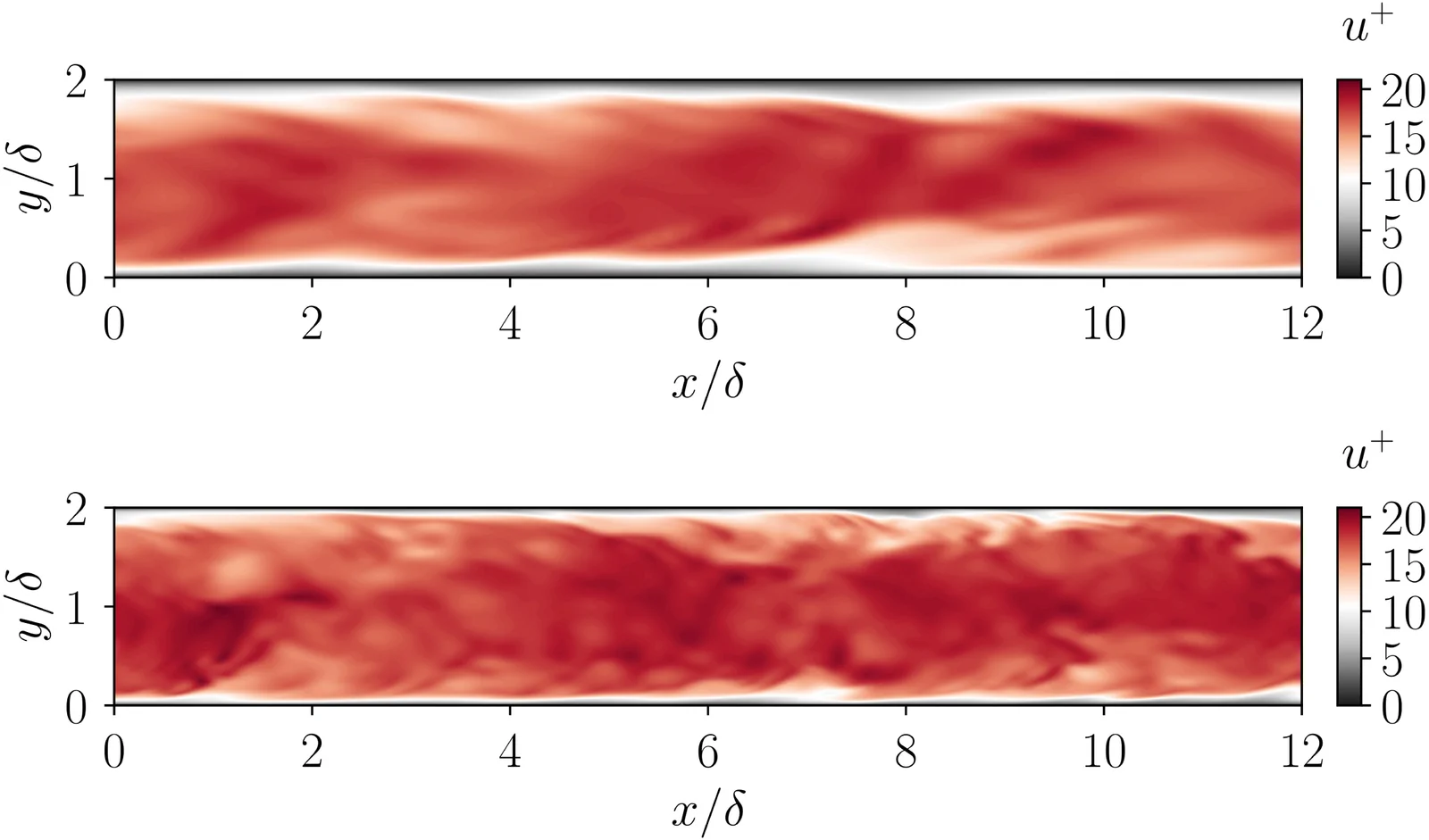
Instantaneous contours of normalized streamwise velocity, $u^{+}$, in the x/δ−y/δ plane for (top) Case I and (bottom) Case II.

**Fig. 4:**
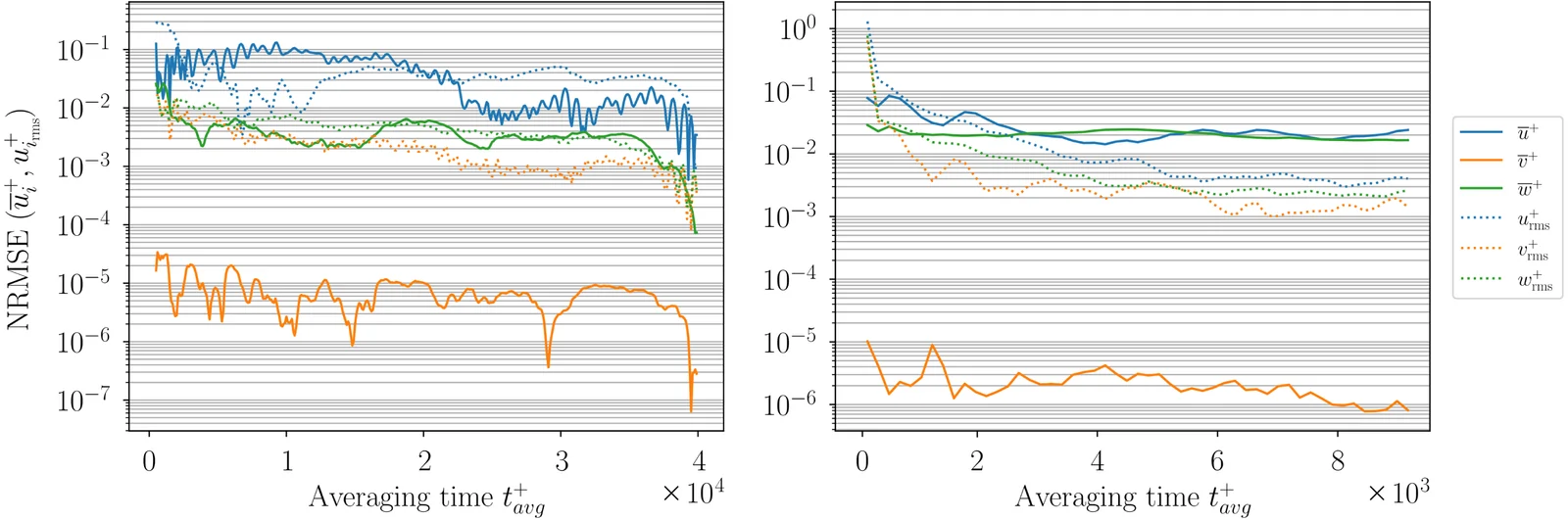
Baseline convergence history for (left) Case I at $Re_{\tau }=100$ and (right) Case II at $Re_{\tau }=180$, with no RL-based control and evaluated using the conservative viscous time step $\Delta t_{c}^{+}$. The normalized root mean square error (NRMSE) of the mean and fluctuating contributions of the velocity field is shown as a function of non-dimensional averaging time, $t_{\text{avg}}^{+}$.

### 4.2 Flow physics environment: Turbulent channel flow

The DRL agents are trained and validated using a DNS of a three-dimensional incompressible turbulent channel flow, a standard benchmark problem for wall-bounded turbulence (Smits et al., 2011). Two simulation configurations are used: Case I at a friction Reynolds number of $Re_{\tau }=100$, and Case II at $Re_{\tau }=180$. The DRL agents are trained exclusively on Case I ($Re_{\tau }=100$) in a supervised manner, and their performance is subsequently evaluated in an unsupervised framework on both Case I, for effectiveness, and Case II, for generalization. The numerical setup for these cases is based on the canonical turbulent channel flow configurations detailed in Chevalier et al. (2006) and Moser et al. (1999). For the low-Reynolds-number canonical channel flow at $Re_{\tau }=100$, the flow remains well within the self-sustained, fully developed regime of wall-bounded turbulence and is clear of marginal or intermittent turbulence behaviors. This is supported by fundamental turbulence studies establishing that the critical threshold for sustaining wall turbulence in channel flows occurs at a much lower limit of $Re_{\tau }\approx 60$ (Jiménez and Moin, 1991, Tsukahara et al., 2005). Furthermore, the corresponding grid resolution summarized in Table 1 ($\Delta x^{+}=19.6,\;\Delta y^{+}=3.1,\;\Delta z^{+}=6.5$) matches the canonical grid constraints validated by Chevalier et al. (2006), demonstrating that a 64 × 64 × 64 grid resolution within this exact domain size is mathematically adequate for successfully resolving dissipative scales. A complete validation of the flow solver against the $Re_{\tau }=180$ reference data is provided in Appendix A.

As illustrated in Fig. 2, the computational domain is 4πδ×2δ×4/3πδ in the streamwise (*x*), wall-normal (*y*), and spanwise (*z*) directions, respectively. The streamwise and spanwise boundaries are set periodic, and no-slip conditions are imposed on the horizontal boundaries (*x*-*z* planes). For both cases, a constant mass flow rate is maintained via a proportional feedback control on the streamwise body force to keep the target $Re_{\tau }$ constant.

The simulation is initialized with a linear velocity profile superimposed with random fluctuations (Nelson & Fringer, 2017). The system is evolved in time until fully-developed turbulent flow conditions are achieved. All relevant physical and numerical parameters are summarized in Table 1.

Baseline simulations of both cases are run without REP-DRL control until a statistically steady state is achieved to establish a ground truth for training and evaluation. These baseline DNS runs are executed using the conservative viscous time step $\Delta t_{c}^{+}$ detailed in Table 1. The flow statistics are then averaged for extended periods, $t_{{\text{avg}}_{\text{C}}}$, corresponding to approximately 400 FTTs for Case I and 173 FTTs for Case II, where one flow-through time (FTT) is defined as $t_{\text{FTT}}=L_{x}/u_{b}∼\delta /u_{\tau }$ with $L_{x}=4\pi \delta$ and the bulk velocity $u_{b}$ estimated using the empirical power-law approximation $Re_{b}=\rho u_{b}2\delta /\mu \approx {\left(Re_{\tau }/0.09\right)}^{1/0.88}$ (Pope, 2000). The resulting converged data provides the reference solution for the subsequent RL training and performance analysis. Instantaneous snapshots of the streamwise velocity fields, depicted in Fig. 3, qualitatively illustrate the effect of the increased Reynolds number. The flow in Case II ($Re_{\tau }=180$) exhibits a wider range of turbulent scales, with more intense velocity fluctuations, particularly in the near-wall region, compared to the smoother structures observed in Case I ($Re_{\tau }=100$).

The quantitative convergence histories of these baseline simulations are presented in Fig. 4, revealing distinct convergence behaviors for each case. The lower-Reynolds number Case I (left) demonstrates a slow decay of the convergence error, a process significantly hindered by persistent, large-amplitude oscillations with a period of approximately $t^{+}=10^{3}$. These oscillations are associated with large-scale streamwise-coherent structures typical of wall-bounded turbulence, whose wavelengths are supported within the employed periodic streamwise and spanwise domain of sufficient size (4πδ×2δ×(4/3)πδ), as detailed in Table 1. These low-frequency dynamics are particularly pronounced in the streamwise velocity component (u), and constitute a limiting factor for rapid statistical convergence due to their long temporal correlation times. In contrast, the higher-Reynolds number Case II (right) exhibits a much faster initial reduction in statistical error and is free of the large-scale oscillations that dominate Case I. While an increased Reynolds number typically extends convergence times due to a greater separation of scales, the dominant factor here is a fundamental change in the flow’s structure. At $Re_{\tau }=180$, the broader inertial range of the more developed turbulence disrupts the formation of the large, coherent structures responsible for the oscillations at the lower Reynolds number. This results in a more rapid statistical de-correlation of the flow’s temporal history and, counter-intuitively, a faster convergence time.

### 4.3 Markov decision process formulation

The optimal control problem is formulated as a MDP, a standard mathematical framework for modeling decision-making in sequential decision problems (Puterman, 2014). This framework defines the closed-loop interaction between the learning agents and the environment, which in this context is the DNS simulation.

#### 4.3.1 Agent distribution & agent-environment interaction

To apply this framework to the DNS environment, a multi-agent approach is employed to implement RL-based $R_{ij}$ perturbations across distinct flow regimes. Agents are uniformly distributed throughout the computational domain $\Omega =L_{x}\times L_{y}\times L_{z}$, forming a three-dimensional multi-agent grid of $N_{a}=N_{a_{x}}\times N_{a_{y}}\times N_{a_{z}}$ agents. The position of agent $a_{ijk}$ is given by its grid point centroid, also referred to as the control node, given by

(19) $$x_{ijk}=\left[\left(i+\frac{1}{2}\right)\Delta x,\;\left(j+\frac{1}{2}\right)\Delta y,\;\left(k+\frac{1}{2}\right)\Delta z\right],$$

where the grid spacings of the agents are $\Delta x=L_{x}/N_{a_{x}}$, $\Delta y=L_{y}/N_{a_{y}}$, and $\Delta z=L_{z}/N_{a_{z}}$, and the indices range as $i=0,\ldots ,N_{a_{x}}-1$, $j=0,\ldots ,N_{a_{y}}-1$, and $k=0,\ldots ,N_{a_{z}}-1$. For compactness, the multi-index ijk is hereafter replaced by a linear index n, defined as

(20) $$n=i+j\;N_{a_{x}}+k\;N_{a_{x}}N_{a_{y}},\;n=0,\ldots ,N_{a}-1,$$

with $N_{a}=N_{a_{x}}N_{a_{y}}N_{a_{z}}$ denoting the total number of agents. Thus, each agent $a_{n}$ interacts with its corresponding subdomain $D_{n}$, defined as the agent centered at the control node $x_{n}$, expressed as

(21) $$D_{n}=\left\{x\in \Omega \;\;|\;\;{\left(x_{i}\right)}_{n}-\frac{\Delta x_{i}}{2}\leq x_{i}<{\left(x_{i}\right)}_{n}+\frac{\Delta x_{i}}{2},\;\text{for}\;\;i=0,1,2\;\right\}.$$

To streamline computational efficiency and simplify implementation, the MPI-based domain decomposition follows the same spatial partitioning as the agent grid. This ensures a one-to-one correspondence between computational subdomains and RL agents.

The MDP interaction loop between the DNS environment and the agents proceeds as follows. At each control step m, the DNS environment provides each agent n with an observation $s_{n}^{m}$, often referred to as the state, which encodes relevant turbulence statistics of its subdomain $D_{n}$. Based on this observation, the agent’s policy $\pi \left(a_{n}^{m}|s_{n}^{m}\right)$ selects an action $a_{n}^{m}$, which determines the corresponding forcing term ${\left(f_{\text{REP}}\right)}_{n}^{m}$. The simulation is then advanced for one actuation period, $t_{\text{act}}$, under this forcing. Finally, at step m+1, the simulation returns a new observation $s_{n}^{m+1}$ and a scalar reward $r_{n}^{m}$. This reward quantifies the effectiveness of the action $a_{n}^{m}$ taken in $s_{m}$, which itself is influenced by both previous actions and actions from neighboring spatial subdomains.

This interaction cycle, represented by the experience tuple generically denoted as $\left(s,a,r,s^{\prime }\right)$, is repeated until a termination criterion is reached, e.g., maximum simulation duration or specific converge criterion, completing an episode. The sequence of collected experiences forms a trajectory

(22) $$\tau =\left[\left(s^{0},a^{0},r^{0},s^{1}\right),\left(s^{1},a^{1},r^{1},s^{2}\right),\;\ldots \;\right].$$

**Fig. 5:**
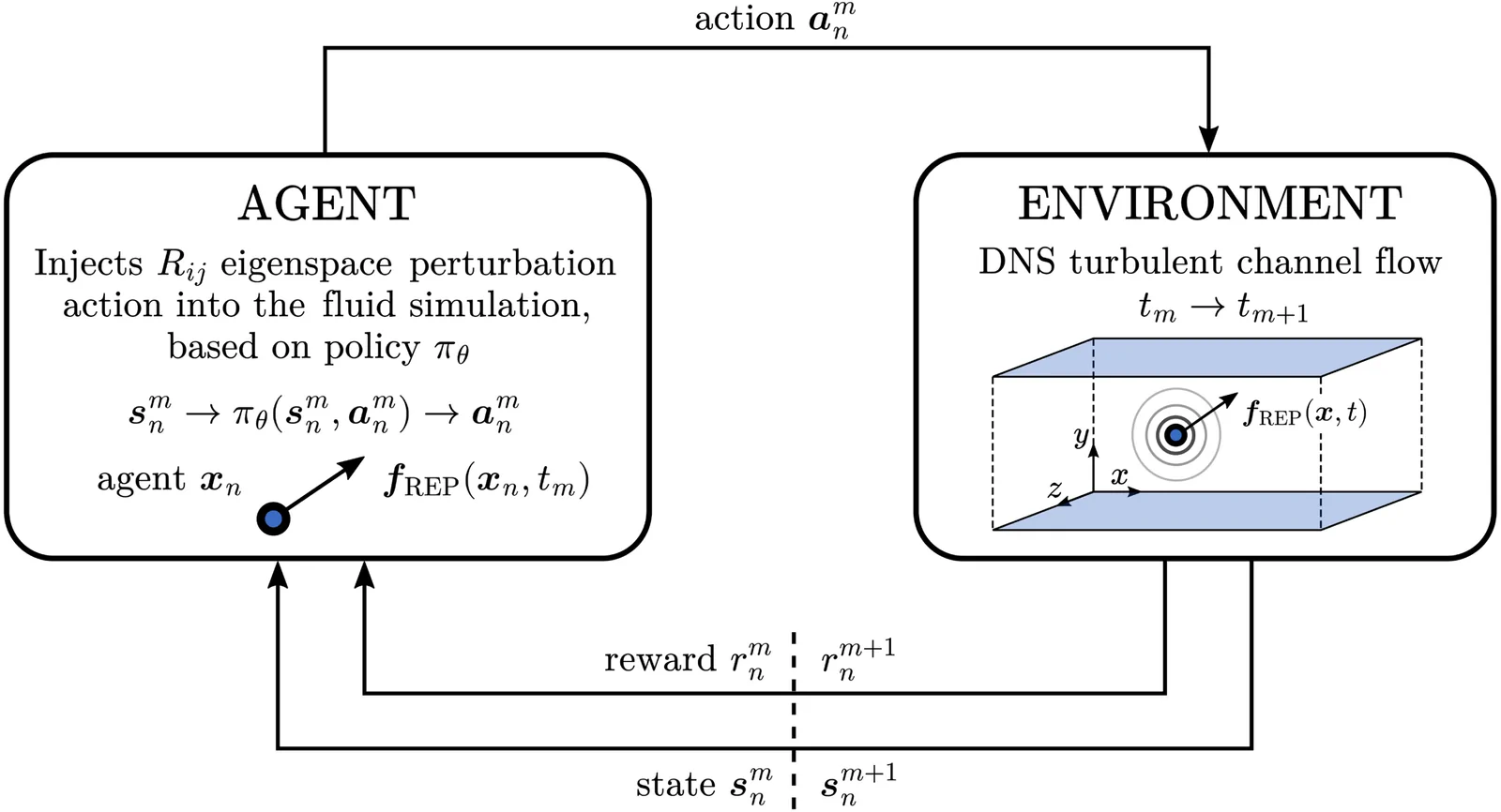
Reinforcement learning control loop for the statistics convergence acceleration framework of a DNS turbulent channel flow.

The objective of the REP-DRL framework is to optimize the control policy π(a|s) to maximize the expected cumulative reward, formally known as the return. To achieve this, agents often learn a value function V(s), which estimates this expected return for a particular state s. By learning an accurate value function, the agent can be trained to select actions that lead to high-return states, thereby discovering the control strategy that achieves the fastest statistical convergence. This complete interaction loop is illustrated in Fig. 5.

#### 4.3.2 State space

The state vector $s_{n}^{m}$ for agent n at step m is formulated from volume-averaged flow quantities within its subdomain $D_{n}$ as

(23) $$f_{n}^{m}=\frac{1}{V_{n}}\int_{D_{n}}f\left(x,t_{m}\right)\;dV,$$

where $V_{n}=\int_{D_{n}}\;dV$ is the volume of the agent subdomain. Additionally, field deviations from a reference state $f_{r}\left(x\right)=f\left(x,t_{r}\right)$ are quantified using the normalized root mean squared error (NRMSE) given by

(24) $$E_{n}^{m}\left(f\right)=\frac{\sqrt{\int_{D_{n}}{\left(f\left(x,t_{m}\right)-f_{r}\left(x\right)\right)}^{2}\;dV}}{\sqrt{\int_{D_{n}}f_{r}{\left(x\right)}^{2}\;dV}}.$$

Two reference strategies for $t_{r}$ are considered, leading to distinct formulations of this metric. In the supervised approach, $t_{r}=t_{C}$ corresponds to a statistically converged flow state, thereby guiding the policy learning toward reproducing this target distribution. Conversely, the unsupervised approach uses $t_{r}=t_{m-1}$, the simulation time of the previous RL step, providing information about the system’s convergence toward a statistically stationary regime without relying on a predefined baseline state. Both strategies promote convergence of flow statistics, but the supervised formulation explicitly enforces agreement with a known baseline solution, whereas the unsupervised approach focuses on statistical stationarity.

Building on this metric, the multi-dimensional state vector $s_{n}^{m}$ encodes selected flow statistics that inform the control policy. Therefore, effective policy learning depends critically on the design of the state vector, which must sufficiently represent the relevant flow characteristics. Two distinct state formulations are considered: (i) a velocity-based state, and (ii) a velocity-error-based state defined as

(25) $$\text{(i)}\;s_{n}^{m}=\left[\;{\left({\bar{u}}^{+}\right)}_{n}^{m},\;{\left({\bar{v}}^{+}\right)}_{n}^{m},\;{\left({\bar{w}}^{+}\right)}_{n}^{m},\;{\left(u_{\text{rms}}^{+}\right)}_{n}^{m},\;{\left(v_{\text{rms}}^{+}\right)}_{n}^{m},\;{\left(w_{\text{rms}}^{+}\right)}_{n}^{m},\;x/L_{x},\;y/L_{y},\;z/L_{z}\;\right],$$

(26) $$\text{(ii)}\;s_{n}^{m}=\left[\;E_{n}^{m}\left({\bar{u}}^{+}\right),\;E_{n}^{m}\left({\bar{v}}^{+}\right),\;E_{n}^{m}\left({\bar{w}}^{+}\right),\;E_{n}^{m}\left(u_{\text{rms}}^{+}\right),\;E_{n}^{m}\left(v_{\text{rms}}^{+}\right),\;E_{n}^{m}\left(w_{\text{rms}}^{+}\right),\;x/L_{x},\;y/L_{y},\;z/L_{z}\;\right],$$

where $\bar{\left(\cdot \right)}$ denotes the mean, subscript ${\left(\cdot \right)}_{\text{rms}}$ corresponds to root-mean-square fluctuations, and superscript ＋ indicates normalization in wall units. Both representations are augmented with the agent’s normalized coordinates.

Although the proposed state formulations do not constitute a complete description of the turbulent flow field, they provide a compact and computationally tractable representation of the local target statistics suitable for this proof-of-concept study. Furthermore, the velocity-error-based state formulation [Eq. (26)] is not solely determined by flow statistics at the current time $t_{m}$, but implicitly incorporates temporal information through deviations with respect to a reference state at $t_{r}$. Depending on the operating strategy, this reference flow state corresponds either to the converged statistics ($t_{r}=t_{C}$) or to the previous control step ($t_{r}=t_{m-1}$). Preliminary experiments were also conducted using an augmented observation containing the five previous local states of each agent; however, no measurable improvement in training performance was observed. Consequently, the present study retains the simpler state definitions of Eqs. (25), (26), while more advanced memory-based architectures and state representations remain directions for future research (see Section 6).

#### 4.3.3 Action space & smoothing

The action $a_{n}^{m}$ selected by the policy is a multi-dimensional, dimensionless vector output by the policy neural network, compactly expressed as

(27) $$a_{n}^{m}=\left[\;{\left(a_{k}\right)}_{n}^{m},\;{\left(a_{x_{1}}\right)}_{n}^{m},\;{\left(a_{x_{2}}\right)}_{n}^{m},\;{\left(a_{\phi_{1}}\right)}_{n}^{m},\;{\left(a_{\phi_{2}}\right)}_{n}^{m},\;{\left(a_{\phi_{3}}\right)}_{n}^{m}\;\right],$$

where each component $a_{\alpha }$ is normalized to the range [−1,1] by the policy network’s output activation function. This normalized action is then scaled to compute the physical eigenspace perturbation vector ${\left(\Delta q\right)}_{n}^{m}$ as

(28) $${\left(\Delta q\right)}_{n}^{m}=k\;\;⊙\;\;a_{n}^{m},$$

where $k=\left[k_{k},\;k_{x_{1}},\;k_{x_{2}},\;k_{\phi_{1}},\;k_{\phi_{2}},\;k_{\phi_{3}}\right]$ is a vector of scaling factors that define the maximum perturbation for each eigenspace parameter, and ⊙ denotes the element-wise product. As detailed in Section 3, this perturbation vector is added to the unperturbed state $q_{n}^{m}$ to obtain the final perturbed eigenspace vector ${\left(\overset{\sim }{q}\right)}_{n}^{m}=q_{n}^{m}+{\left(\Delta q\right)}_{n}^{m}$. From this, the corresponding perturbed tensor ${\left(R_{ij}\right)}_{n}^{m}$, the perturbation tensor ${\left(\Delta R_{ij}\right)}_{n}^{m}$, and the resulting REP-based forcing term ${\left(f_{\text{REP}}\right)}_{n}^{m}$ are computed for agent n and step m.

A critical aspect of the implementation is the spatial treatment of the forcing term. The eigenspace decomposition is performed only at the agent’s control node, $x_{n}$, meaning the perturbation ${\left(\Delta R_{ij}\right)}_{n}^{m}$ is only known at each discrete points. To compute the volumetric forcing $f_{\text{REP}}$ from these control nodes, the partial spatial derivative $\partial \Delta R_{ij}/\partial x_{j}$, required by (18), must be approximated. This is achieved by assuming $\Delta R_{ij}$ linearity between adjacent agent control nodes and employing second-order finite differences. This approximation results in a REP-based forcing term $f_{\text{REP}}$ that is piecewise-constant in space.

Applying this forcing in a temporally discrete manner can lead to numerical instabilities. To enforce temporal continuity and ensure a time-correlated signal, the perturbation forcing is interpolated between consecutive control steps $t_{m-1}$ and $t_{m}=t_{m-1}+t_{\text{act}}$ as (Rabault et al., 2019)

(29) $$f_{\text{REP}}\left(x,t\right)=f_{\text{REP}}\left(x,t_{m-1}\right)+g_{3}\left[f_{\text{REP}}\left(x,t_{m}\right)-f_{\text{REP}}\left(x,t_{m-1}\right)\;\right],\;\text{for}\;t\in \left(t_{m-1},t_{m}\right),$$

where the temporal smoothing coefficients are defined as

(30) $$g_{1}=e^{-\frac{1}{\xi }},\;g_{2}=e^{-\frac{1}{1-\xi }},\;g_{3}=\frac{g_{1}}{g_{1}+g_{2}},$$

with ξ the normalized actuation time, expressed as

(31) $$\xi =\frac{t-t_{m-1}}{t_{\text{act}}},\;\text{for}\;t\in \left(t_{m-1},t_{m}\right).$$

#### 4.3.4 Reward function

The scalar reward $r_{n}^{m}$, associated with agent n and control step m, provides feedback on the convergence of targeted statistical quantities. This is computed based on the NRMSE (24) of first- and second-order velocity statistics as

(32) $$r_{n}^{m}=b-[c_{1}E_{n}^{m}\left({\bar{u}}^{+}\right)+c_{2}E_{n}^{m}\left({\bar{v}}^{+}\right)+c_{3}E_{n}^{m}\left({\bar{w}}^{+}\right)+c_{4}E_{n}^{m}\left(u_{\text{rms}}^{+}\right)+c_{5}E_{n}^{m}\left(v_{\text{rms}}^{+}\right)+c_{6}E_{n}^{m}\left(w_{\text{rms}}^{+}\right)+c_{7}\|{\left(f_{\text{REP}}\right)}_{n}^{m}\|],$$

where b and $c=\left[c_{1},\ldots ,c_{7}\right]$ are reward parameters. In this general formulation of the reward function, the b parameter provides a positive baseline reward, the weighting coefficients $c_{1},\ldots ,c_{6}$ are set to balance the relative importance of converging each statistical quantity, and the $c_{7}$ coefficient explicitly penalizes the magnitude of the applied forcing term. In the present work, this forcing penalty is deactivated ($c_{7}=0$) due to the early, short averaging time windows chosen for the numerical experiments, as detailed in Section 5.1.

As detailed in Section 4.3.2, these error terms are calculated with respect to a reference flow state at $t_{r}=t_{C}$ for supervised training, and $t_{r}=t_{m-1}$ for unsupervised evaluation, where the framework operates without prior knowledge of the high-fidelity converged reference statistics. Consequently, the unsupervised reward formulation inherently penalizes the temporal gradients of the targeted flow statistics between subsequent control steps rather than their absolute deviation from true converged statistics at $t_{C}$. As a result, if the control policy drives the fluid system into an altered or shifted statistically stationary state ($s_{n}^{m}\approx s_{n}^{m-1}$), the local unsupervised error metrics will vanish ($E_{n}^{m}\to 0$). Under these conditions, the agent is heavily rewarded for maintaining stationarity, despite the global statistical bias introduced. While this behavior exposes a potential failure mode of the unsupervised reward formulation, it also reflects the absence of converged reference statistics during deployment. Given this constraint, the temporal-gradient-based error strategy is found to be the most effective and stable approach found for driving flow statistics to convergence in the unsupervised framework.

### 4.4 Algorithm selection: Proximal policy optimization

The MDP defined above presents a significant challenge: finding the optimal control policy $\pi^{⋆}$ in a high-dimensional, continuous space that maximizes the expected return. Standard, tabular RL methods attempt to solve this by representing the policy or value functions as a lookup table, which is only feasible for problems with small, discrete state spaces. This problem, however, features a high-dimensional, nonlinear and continuous state space, as the observations $s_{n}^{m}$ are composed of continuous turbulence statistics. To overcome this challenge, function approximation is required, which allows the agent to generalize from experienced to unobserved states by learning a parameterized function. DRL is a modern approach that directly addresses this problem by employing deep neural networks (DNNs) as powerful, nonlinear function approximators (Arulkumaran et al., 2017, Mnih et al., 2015, Sutton and Barto, 2018). In this framework, DNNs are used to learn parameterized representations of essential functions governing the agent’s behavior. These can include the policy π(a|s,θ), which maps states to actions; the value function $V^{\pi }\left(s,w\right)$ or the Q-function $Q^{\pi }\left(s,a,w\right)$, which estimates expected returns; or the environment model $P\left(s^{\prime }|s,a,\phi \right)$, which predicts state transition probabilities.

The selection of a specific DRL algorithm is guided by key constraints imposed by the problem. Primarily, DRL algorithms are broadly classified as model-based and model-free approaches. Model-based algorithms learn a predictive model of the environment dynamics, $P\left(s^{\prime }|s,a\right)$, an objective that is computationally prohibitive for the turbulent flow dynamics in this work. Therefore, a model-free algorithm, which learns a control policy directly from experience, is required. Second, model-free algorithms are further classified as value-based and policy-based depending on the specific function to optimize. Value-based methods, including deep Q-learning (DQN) (Mnih et al., 2013) and its extensions, learn only a value function and are designed for discrete action spaces, selecting an action by finding the maximum Q-value over a finite set of discrete actions. This optimization operation is computationally expensive and poorly adapted to continuous control tasks. Given that this problem features a continuous and multi-dimensional action space, i.e., the six-component Δq vector, value-based methods are rendered unsuitable. This constraint requires a policy-based approach, which directly parametrizes the policy π(a|s,θ) to output continuous values. Specifically, this work employs an actor-critic method, a hybrid method that is a sub-class of policy-based methods, as it learns both a policy (the actor π) and a value function (the critic V) to accelerate and stabilize the learning process. Finally, DRL algorithms are categorized as on-policy and off-policy based on how training data is utilized. On-policy algorithms train using data generated by the current policy $\pi_{t}$, discarding this data after each policy update. Off-policy algorithms reuse data generated by previous policies, improving sample efficiency at the cost of increased memory requirements. However, this can lead to training instability, as the agent learns from suboptimal trajectories, especially for complex systems such as the turbulent channel flow in this problem. This leads to the selection of an on-policy algorithm for this work.

These considerations (viz. the need for a model-free, policy-based, on-policy algorithm capable of handling continuous observation and action spaces) motivate the use of PPO (Schulman et al., 2017). PPO is a state-of-the-art algorithm within the family of policy gradient (PG) methods, which optimizes the parameters of two separate neural networks: the actor, given by the policy network π(a|s,θ); and the critic, given by the value function $V^{\pi }\left(s,w\right)$. The choice of PPO is motivated by its ability to solve two major challenges inherent in standard on-policy algorithms. First, traditional PG algorithms, such as REINFORCE (Williams, 1992) and standard Actor-Critic (Konda & Tsitsiklis, 2003), often suffer from high-variance in the gradient estimates, leading to large policy updates and subsequent performance collapse. Moreover, on-policy algorithms generally suffer from poor sample efficiency, as an entire batch of experience is discarded after only one gradient update. This limits their applicability in computationally expensive environments, such as the DNS in this work.

PPO addresses these challenges by building on its predecessor, trust region policy optimization (TRPO) (Schulman, Levine, et al., 2015). To ensure stability, PPO introduces a clipped surrogate objective function that penalizes large policy updates, thereby ensuring monotonic policy improvement while avoiding the computational overhead associated with TRPO’s trust region constraints. Additionally, PPO enhances sample efficiency by employing minibatch training and allowing limited data reuse within its on-policy framework, thus mitigating some of the inefficiencies of traditional on-policy algorithms. This combination of training stability, implementation simplicity, and improved sample efficiency makes PPO particularly well-suited for optimizing control policies in computationally costly environments. For further details, brief introductions to the PG method and the PPO algorithm are provided in Appendix B and Appendix C, respectively.

## 5 Numerical experiments & discussion

This section presents the preliminary numerical results from the exploration of the proposed REP-DRL framework, as a proof-of-concept designed to assess its potential for accelerating the convergence of turbulent flow statistics. The analysis begins by establishing the general computational setup for training and evaluation, including the key PPO hyper-parameters. Next, the results of the supervised training process on the low-Reynolds number ($Re_{\tau }=100$) case are analyzed, which provides insight into the policy’s learning dynamics and the selection of an optimal policy. The performance of the selected optimal policy is then evaluated in an unsupervised setting, first on the $Re_{\tau }=100$ training case to explore its behavior, and then on a higher-Reynolds number ($Re_{\tau }=180$) case to evaluate its adaptability to an unseen flow regime. Furthermore, a sensitivity analysis for the actuation period is presented to further investigate the policy’s sensitivity and operational range. Finally, a comprehensive computational cost and amortization analysis is performed to evaluate the REP-DRL framework’s practical viability, determining the exact number of deployment runs required to fully recover the initial $Re_{\tau }=100$ training overhead when accelerating the convergence of fluctuating velocity components at $Re_{\tau }=180$.

### 5.1 Training & evaluation setup

The present study is designed as a proof-of-concept study conducted in a simplified and computationally tractable turbulent channel flow configuration at low friction Reynolds numbers ($Re_{\tau }=100$ and 180), enabling the assessment of the learning dynamics and control effectiveness under identical geometric, numerical, initialization, and training conditions. Therefore, this setting is intentionally restricted to a low-Reynolds-number regime in order to clearly analyze the REP-DRL learning and actuation mechanisms in a controlled numerical environment. Nevertheless, despite ensuring interpretability and computational feasibility, this design choice limits the present study to a low-Reynolds-number regime that does not capture the full complexity of high-Reynolds-number turbulent flows targeted in practical applications, as further discussed in Section 6.

All DNS baseline simulations in Section 4.2, DRL training episodes in Section 5.2, and policy evaluation deployments in Sections 5.3, 5.4 are executed using the conservative time steps ($\Delta t_{c}^{+}$) listed in Table 1. This conservative temporal discretization is initially selected for code simplicity during implementation, guaranteeing that the control actuation period ($t_{\text{act}}^{+}$) remains an exact integer multiple of the DNS integration step ($N_{c}=t_{\text{act}}^{+}/\Delta t_{c}^{+}=500$ across all cases as detailed in Table 2). Crucially, the actuation period $t_{\text{act}}^{+}$ is an explicit physical timescale of the control framework, defined independently of the underlying DNS numerical integration step $\Delta t^{+}$.

The PPO algorithm introduced in Section 4.4 is implemented within an episode-based framework. To enhance computational efficiency, all training episodes are initialized from a fully-developed turbulent state, achieved by advancing the simulation without active flow control until turbulent steady-state conditions are reached. This flow state, which is turbulent but not yet statistically converged, is then averaged over a short time interval $t_{{\text{avg}}_{0}}$, and saved to be used as the initial condition for all subsequent training episodes. This approach avoids the computational overhead associated with both re-running the simulation’s initial transient phase and repeating the preliminary averaging period $t_{{\text{avg}}_{0}}$ for every episode.

During each training episode, the agents interact with this fully developed turbulent flow for a simulation duration of $t_{\text{ep}}$, collecting a trajectory of $\left(s,a,r,s^{\prime }\right)$ experiences. Once the episode is complete, this collected trajectory data is used to optimize the policy and value networks via mini-batch gradient ascent. The updated networks then guide the agents’ actions in subsequent episodes, enabling an iterative refinement loop that optimizes the REP-DRL control strategy to maximize cumulative rewards.

**Table 2:** REP-DRL and PPO parameters.

Parameter	Value/Description
*Environment-Agent Setup*	
Control time step or Actuation period, $t_{\text{act}}^{+}$	Case I: 5; Case II: 4.5
Numerical time steps per actuation period, $t_{\text{act}}^{+}/\Delta t_{c}^{+}$	Case I: 500; Case II: 500
Training episode duration, $t_{\text{ep}}^{+}$	Case I: 100
Evaluation simulation duration, $t_{\text{eval}}^{+}$	Case I: 200; Case II: 180
Initial conditions pre-averaging period, $t_{{\text{avg}}_{0}}^{+}$	Case I: 500; Case II: 180
Number of agents, $N_{a}$	160
Agents distribution, $N_{a_{x}}\times N_{a_{y}}\times N_{a_{z}}$	8×10×2
Batch size	160 (set as $N_{a}$)
Number of training episodes, $N_{\text{ep}}$	100
Optimizer	Adam, learning rate $10^{-3}$
State dimension	9
Action dimension	3 ($k,x_{1},x_{2}$ perturbation)
Action scaling vector, k	[20,5,5]
Reward parameter b	1
Reward parameter c	$\left[\;0.1,0.04,0.04,0.02,0.02,0.02,0.0\;\right]\;/t_{\text{act}}$
Relaxation factor, α	1.0

*DNNs Architecture*	
Policy network	Fully-connected [9,128,128,3]
Value function network	Fully-connected [9,128,128,1]

*PPO Algorithm hyper-parameters*	
Importance ratio clipping, ϵ	0.2
GAE factor, λ	0.95
Discount factor, γ	0.99
Rewards normalization	True
Observations normalization	True
Entropy coefficient	$10^{-3}$
L2-reg. of policy unshared weights	$10^{-5}$
L2-reg. of value function unshared weights	$10^{-5}$
L2-reg. of policy & value shared weights	$10^{-5}$
Value function coefficient	$10^{-3}$

The training process is *supervised* in the sense that the state and reward functions, defined in Section 4, are calculated relative to a final, unperturbed, statistically converged baseline at $t_{\text{C}}$ (with statistical fields averaged over $t_{{\text{avg}}_{\text{C}}}$). To maintain consistency in global performance benchmarking, the normalized root-mean square error (NRMSE) used to monitor both training and evaluation stages is always calculated with respect to this same high-fidelity reference state ($t_{{\text{avg}}_{\text{C}}}$). However, this shared reference does not imply any coupling between the training and evaluation processes. Notably, the supervised training analysis in Section 5.2 and the unsupervised evaluation results in Sections 5.3, 5.4 are therefore based on distinct simulation trajectories. Therefore, while the identification of a representative training checkpoint (episode 17) is performed based on the supervised training analysis, all performance metrics reported in the subsequent evaluation subsections are obtained from independent, unsupervised deployment simulations executed after training completion.

It should be explicitly noted that the supervised training analysis, policy checkpoint selection, and evaluation metrics presented in this section are derived from a single training trajectory using a single initial training seed. Given the substantial computational cost of training multi-agent DRL directly within DNS environments, an exhaustive multi-seed ensemble study remains beyond the scope of this proof-of-concept implementation and is left for future work. Because DRL algorithms such as PPO are subject to across-seed variance (Schulman et al., 2017), the training, deployment and cost amortization results reported herein represent deterministic outcomes of a representative proof-of-concept training run rather than ensemble-averaged statistical results.

Effective training of the PPO algorithm relies on appropriate network architectures and carefully tuned hyper-parameters, which must balance exploration and exploitation for stable policy convergence. In particular, some critical training parameters are the learning rate, discount factor, exploration rate, neural network architecture, and batch size. The main setup parameters and PPO hyper-parameters defining the experimental training and evaluation process are summarized in Table 2.

Based on the parameters in this table, the multi-agent grid of 8 × 10 × 2 results in actuator spacing of $\Delta x_{a}=\pi \delta /2$, $\Delta y_{a}=\delta /5$, and $\Delta z_{a}=2/3\pi \delta$ [Eq. (19)]. While a formal sensitivity analysis with respect to the multi-agent grid resolution ($N_{a_{x}}\times N_{a_{y}}\times N_{a_{z}}$) has not been performed due to the severe computational overhead of training distinct multi-agent DRL frameworks from scratch, this specific agent distribution is designed based on two fundamental criteria to balance the piecewise-constant nature of the gradient-based forcing, $f_{\text{REP}}$, with the underlying physics of a wall-bounded turbulent channel flow. First, to accommodate the anisotropy of wall-bounded turbulence and the elongated domain aspect ratio ($L_{x}\gg L_{y},L_{z}$), a coarser agent resolution is assigned to the periodic streamwise and spanwise directions ($\Delta x_{a}=\pi \delta /2$ and $\Delta z_{a}=2/3\pi \delta$). Conversely, the finer wall-normal distribution ($N_{a_{y}}=10$, yielding $\Delta y_{a}=\delta /5$) targets the steep gradients and complex turbulent structures near the walls. For the symmetric uniform agent distribution employed, the agents are located within control planes at y/δ=[0.1,0.3,0.5,0.7,0.9], which in viscous units corresponds to $y^{+}=\left[10,30,50,70,90\right]$ for $Re_{\tau }=100$, and $y^{+}=\left[18,54,90,126,162\right]$ for $Re_{\tau }=180$. This distribution strategically positions the control agents across the buffer layer and outer flow region, allowing the framework to execute distinct, layer-specific flow perturbations. Second, as the forcing magnitude scales inversely with agent separation ($f_{{\text{REP}}_{i}}^{⋆}\propto 1/\Delta x_{a,j}^{⋆}$), this agent grid density mathematically bounds the spatial derivative. This imposes a numerical constraint to the agents resolution, as an excessively fine agent grid would lead to non-physical, localized step-changes in $f_{\text{REP}}^{⋆}$ that could trigger numerical instabilities in the DNS.

During training, the control time step is defined as $t_{\text{act}}^{+}=5$ and the episode duration is $t_{\text{ep}}^{+}=100$ (corresponding to $N_{c}=t_{\text{act}}^{+}/\Delta t_{c}^{+}=500$ DNS integration steps per actuation period), which results in $t_{\text{ep}}^{+}/t_{\text{act}}^{+}=20$ action updates per training episode. With $N_{a}=160$ agents across the simulation domain, this yields 160 trajectories and 3200 experiences per training episode. Using a batch size equal to the number of agents, this results in 20 policy updates per training episode. In contrast, the evaluation simulation is extended as $t_{\text{eval}}^{+}=180$ with $t_{\text{act}}^{+}=4.5$ (likewise maintaining $N_{c}=t_{\text{act}}^{+}/\Delta t_{c}^{+}=500$ integration steps per actuation period for Case II), i.e., $t_{\text{eval}}^{+}/t_{\text{act}}^{+}=40$ action updates over the evaluation simulation, doubling the duration of the training episodes to better assess long-term stability.

Crucially, these training and evaluation operational windows are intentionally designed to accelerate the convergence of flow statistics at early stages, where the uncontrolled baseline statistics remain far from convergence. To fully eliminate high-order turbulent fluctuations, an uncontrolled baseline simulation typically requires massive integration windows spanning thousands of viscous time units ($t^{+}∼10^{3}$ to $10^{4}$) even at these low friction Reynolds numbers ($Re_{\tau }\in \left[100,180\right]$) (Oliver et al., 2014, Vreman and Kuerten, 2014). In contrast, the present framework operates strictly within highly constrained averaging windows ($t^{+}∼10^{2}$). Specifically, the training configuration for Case I ($Re_{\tau }=100$) reaches a cumulative averaging time of $t_{\text{avg}}^{+}=600$ at the end of each episode, comprising a pre-averaging window of $t_{{\text{avg}}_{0}}^{+}=500$ and an active episode duration of $t_{\text{ep}}^{+}=100$. During the subsequent performance evaluation of Case I ($Re_{\tau }=100$), the simulation employs an extended window of $t_{{\text{avg}}_{\text{E}}}^{+}=200$, which results in a total averaging window of $t_{\text{avg}}^{+}=700$. For the Case II ($Re_{\tau }=180$) generalization test, evaluation begins from a highly unconverged restart field, pre-averaged for only $t_{{\text{avg}}_{0}}^{+}=180$, and extends active flow control for $t_{{\text{avg}}_{\text{E}}}^{+}=180$, concluding at a total averaging window of $t_{\text{avg}}^{+}=360$.

Because the framework operates within these brief, early-stage averaging windows, the asymptotic requirement for $f_{\text{REP}}\to 0$ as the flow statistics approach convergence (Section 3.5) is neither expected nor required during active flow control. Consequently, the direct forcing penalty in the reward function [Eq. (32)] is intentionally disabled by setting $c_{7}=0$. This removes any explicit penalty on action magnitude, enabling the agent to execute high-magnitude $R_{ij}$ perturbations early in the statistics averaging process, allowing active exploration to effectively modify the persistent, slow-converging turbulent structures without stalling convergence acceleration. It should be noted that because $c_{7}=0$, the reward function does not mathematically enforce the attenuation of forcing ($f_{\text{REP}}\to 0$) as the target statistics error becomes small. In short-horizon control windows ($t_{\text{ep}}^{+},t_{\text{eval}}^{+}∼10^{2}$), forcing attenuation is not required as the statistics remain far from convergence. However, for extended integration horizons where full statistics convergence is targeted, reactivating a non-zero forcing penalty ($c_{7}>0$) is required to explicitly drive control actions to zero as the target statistics reach convergence. Furthermore, rather than treating these coefficients as purely empirical hyperparameters requiring exhaustive parametric tuning, their relative magnitudes (c=[0.1,0.04,0.04,0.02,0.02,0.02,0.0]) are established via order-of-magnitude balancing of the reward error tracking terms during preliminary training experiments. Preliminary testing indicates that policy performance remains robust to minor variations in these weights, provided this fundamental balance is preserved, while the constant bias is fixed at b=1 to ensure a positive reward.

Under this paradigm, the role of the applied forcing $f_{\text{REP}}$ is not to act as a localized, high-frequency trap that holds an instantaneous chaotic system in an artificial state, but rather as a macroscopic biasing force. Therefore, by injecting the divergence of the Reynolds stress perturbations directly into the momentum equations, the control policy aims to drive the running time-averaged statistics toward their reference values faster than natural statistical accumulation would allow. However, because a running time-average collected under an active body force ($f_{\text{REP}}\neq 0$) samples forced dynamics rather than the unforced distribution, the resulting convergence acceleration of running statistics is strictly bounded to the active control window. While the bias reduction achieved during active control remains mathematically embedded within the cumulative time-integrated history at the end of the forcing window ($t_{\text{train}}^{+}$ or $t_{\text{eval}}^{+}$), this formulation does not assume that the accelerated statistics persist without relaxation to the unforced dynamics once forcing is deactivated. Once the active control is deactivated ($f_{\text{REP}}=0$), the instantaneous flow naturally returns to its unforced Navier–Stokes dynamics, and subsequent statistical accumulation will most likely cause the running averages to undergo transient physical relaxation back toward the uncontrolled baseline trajectory. These post-actuation continuation simulations under $f_{\text{REP}}=0$ are not conducted in this proof-of-concept study, but quantitatively mapping these statistics relaxation behavior is explicitly identified as an essential direction for future work in Section 6.

As specified in Table 2, a reduced three-dimensional (3-D) action is used for this initial proof-of-concept, which directly perturbs the $R_{ij}$ magnitude and shape with $a_{n}^{m}=\left[\;{\left(a_{k}\right)}_{n}^{m},\;{\left(a_{x_{1}}\right)}_{n}^{m},\;{\left(a_{x_{2}}\right)}_{n}^{m}\;\right]$, instead of the complete six-dimensional (6-D) $R_{ij}$ perturbation action in Eq. (27). By omitting the three Euler angles of the eigenvector matrix, the control policy leaves the spatial orientation of the Reynolds stress eigenvectors unaltered. Physically, this restricts the agent from directly rotating the principal axes of the tensor or manipulating their directional alignment with respect to the mean wall shear gradient $\partial \bar{u}/\partial y$. This eliminates the agent’s ability to explicitly suppress or enhance shear-stress production, thereby bounding the control problem to modulating the amplitude of the velocity fluctuations (turbulence intensity) and their component-wise energy distribution (anisotropy shape). Additionally, to map the normalized outputs of the control policy to physical $R_{ij}$ eigenspace perturbations, the 3-D action employs the scaling vector $k=\left[k_{k},\;k_{x_{1}},\;k_{x_{2}}\right]=\left[20,5,5\right]$, while the static relaxation factor is set to α=1 [Eq. (18)]. These parameters are selected through empirical tuning during preliminary training runs to ensure that the forcing term can rapidly modify slow-evolving, large-scale flow structures despite the limited duration of the training episode.

This reduced 3-D action is highly suited for the canonical, wall-bounded channel flow configuration considered, where the flow is characterized by horizontal spatial homogeneity (x–z planes) and wall-normal mid-plane symmetry across y=δ (as detailed in Section 4.2), ensuring that the principal stress axes remain strongly aligned along the mean shear layer. However, generic non-homogeneous turbulent flows are characterized by severe stress–strain misalignment (such as flows over bluff bodies or separated shear layers), where the full 6-D action space is expected to be required. Mathematically, turbulent kinetic energy production $P_{k}=-\bar{u_{i}^{\prime }u_{j}^{\prime }}S_{ij}$ depends explicitly on the tensor inner product between the Reynolds stress tensor $\bar{u_{i}^{\prime }u_{j}^{\prime }}$ and the mean strain rate tensor $S_{ij}=\frac{1}{2}\left(\partial {\bar{u}}_{i}/\partial x_{j}+\partial {\bar{u}}_{j}/\partial x_{i}\right)$. Therefore, directly manipulating $R_{ij}$ orientation via the full 6-D action space provides a mechanism for regulating turbulence generation efficiently in non-homogeneous flows, enabling comparable or superior performance relative to the reduced 3-D action space. Because DRL algorithms designed for continuous action spaces (such as the PPO algorithm utilized in this work) scale efficiently with action space dimensionality, here requiring only an extension of the policy network output layer from 3 to 6 nodes, utilizing the full 6-D action introduces negligible computational overhead during forward policy execution and backpropagation updates, indicating that the operational viability and efficiency of the framework is expected to extend to higher-dimensional action spaces in complex flow regimes.

Although a direct velocity-based state (25) has been considered, the nine-dimensional (9-D) velocity-error-based state (26) is employed as it is expected to enhance generalization across different $Re_{\tau }$ turbulent cases and to maintain a close resemblance with the error-based reward (32). The state and reward signals are computed using the supervised approach during training (i.e., $t_{r}=t_{C}$) and the unsupervised approach during performance evaluation (i.e., $t_{r}=t_{m-1}$) for the error-based calculations (26). For more details, readers are referred to the code implementation in Appendix D.

### 5.2 Supervised training analysis ($Re_{\tau }=100$)

The supervised training process is conducted on Case I ($Re_{\tau }=100$), as detailed in Section 4.2. The presented training analysis is restricted to diagnostic interpretation of learning dynamics and checkpoint selection within the training process, and does not rely on any evaluation trajectories from Section 5.3 onward. Additionally, to ensure a fair performance comparison, the total simulation time of the REP-DRL training process is directly matched against that of the uncontrolled baseline. This approach is justified because the overall computational expense of the REP-DRL framework is governed almost entirely by the numerical flow solver, while the overhead from DRL operations (including state sensing, reward calculation, and network updates) is negligible.

To achieve this, training is structured into up to $N_{\text{ep}}=100$ episodes. Every training episode resets to the same fully developed turbulent restart field, which is pre-averaged over an initial window of $t_{{\text{avg}}_{0}}^{+}=500$. From this initialized state, each episode advances the flow simulation for a target duration of $t_{\text{ep}}^{+}=100$. However, to ensure training stability, episodes are terminated early if the running time-averaged bulk streamwise velocity ${\bar{u}}_{b}$ deviates by more than 5% from its theoretical value, ${\left({\bar{u}}_{b}\right)}_{\text{th}}$. This target is derived from the bulk Reynolds number approximation $Re_{\tau }\approx 0.09\;Re_{b}^{0.88}$ (Pope, 2000), which defines ${\left({\bar{u}}_{b}\right)}_{\text{th}}=\mu \;Re_{b}/\left(\rho 2\delta \right)$. Consequently, at the end of each training episode, the statistical fields for the REP-DRL controlled case are only averaged over a maximum, constrained window of $t_{\text{avg}}^{+}=t_{{\text{avg}}_{0}}^{+}+t_{\text{ep}}^{+}=600$. Nonetheless, to monitor the learning progression against the total computational cost, the REP-DRL performance metrics at the end of each episode are compared against the baseline results at the equivalent *cumulative* simulation time accumulated across all preceding episodes. This specific training process assessment gives the uninterrupted baseline an increasing statistical averaging advantage over the REP-DRL episodic checkpoints, which explains why the baseline exhibits smaller errors for the fluctuating velocity components in the results presented below.Fig. 6Supervised training performance (REP-DRL control, red) compared to the uncontrolled baseline (blue). The NRMSE of the mean and fluctuating velocity components is plotted against cumulative averaging time ($t_{\text{avg}}^{+}$) for: (a) streamwise (${\bar{u}}^{+}$, $u_{\text{rms}}^{+}$), (b) wall-normal (${\bar{v}}^{+}$, $v_{\text{rms}}^{+}$), and (c) spanwise (${\bar{w}}^{+}$, $w_{\text{rms}}^{+}$) components. (For interpretation of the references to color in this figure legend, the reader is referred to the web version of this article.)Fig. 6

**(a):**
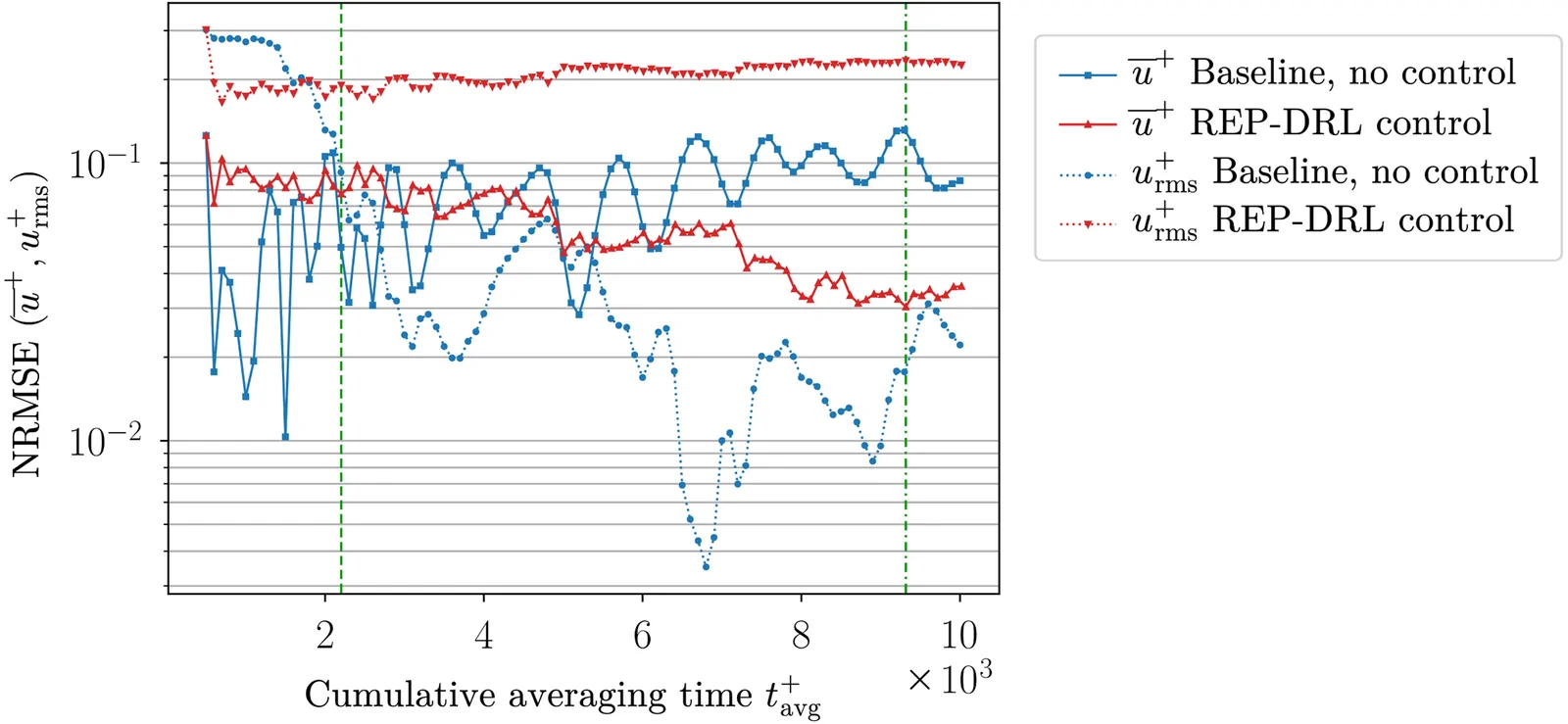
.

**(b):**
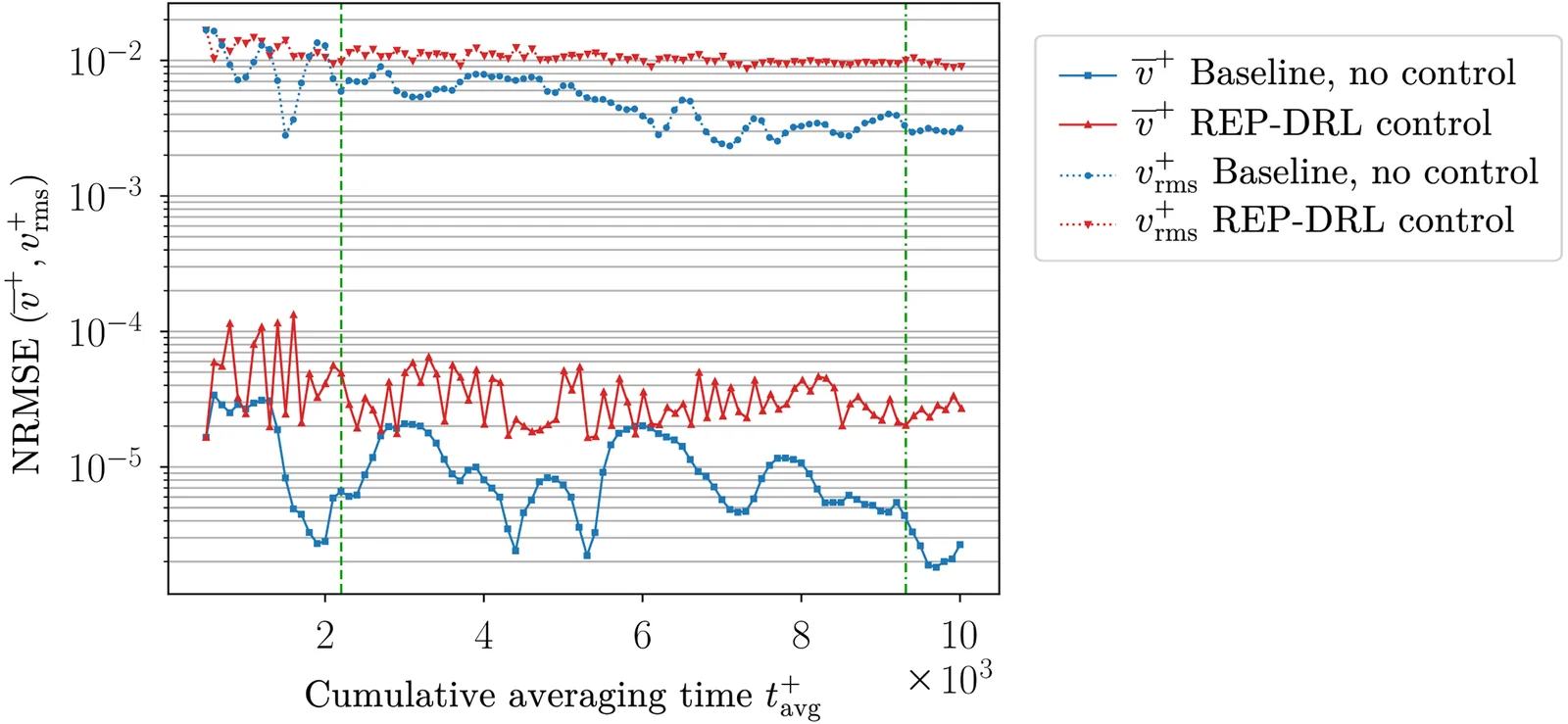
.

**(c):**
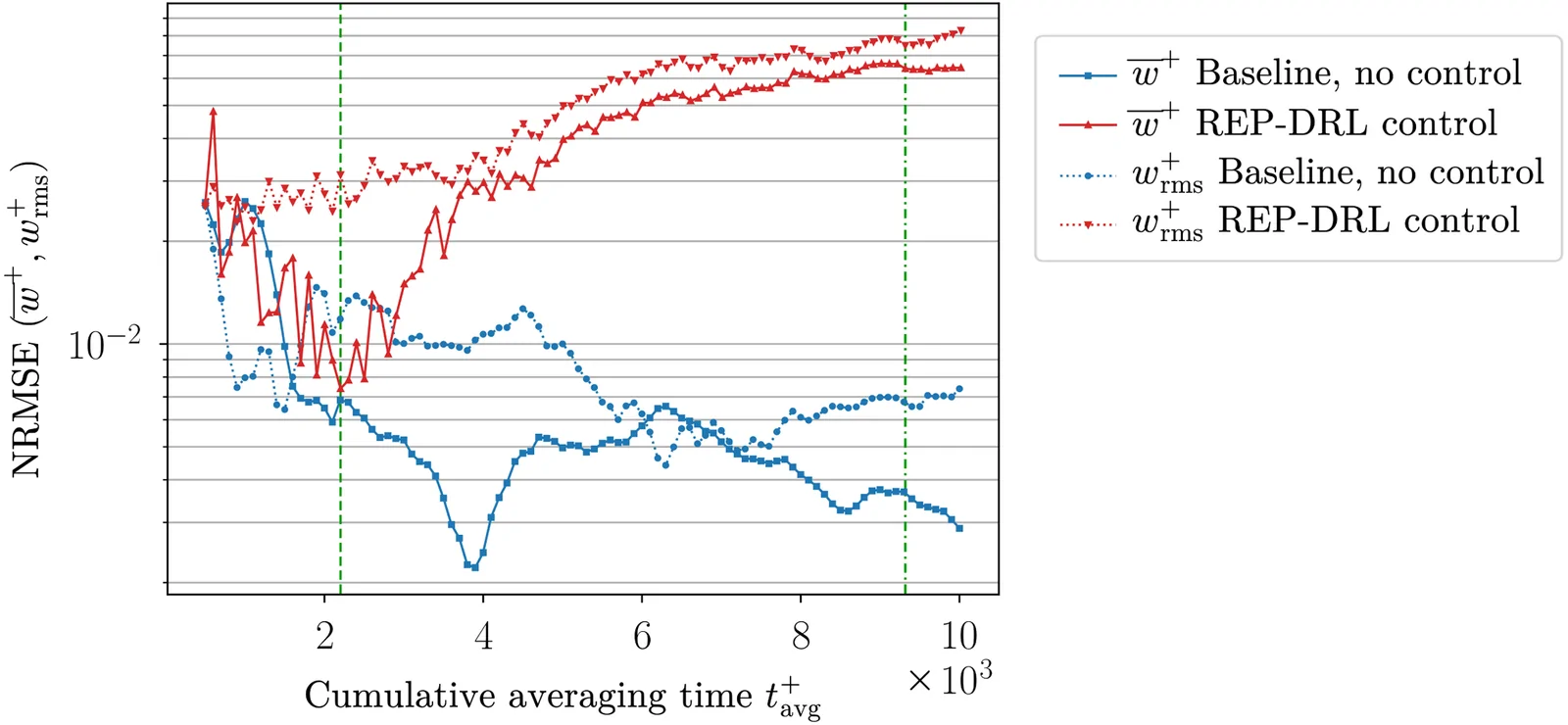
.

Fig. 6 shows the evolution of the velocity statistics error, relative to the converged reference state averaged for $t_{{\text{avg}}_{\text{C}}}$, throughout the entire training process. The supervised NRMSE (24) of evaluated fields is calculated at the end of each training episode, with blue lines corresponding to the uncontrolled baseline at a comparable cumulative $t_{\text{avg}}^{+}$, and red lines representing the REP-DRL control case. In Fig. 6(a), the uncontrolled baseline exhibits the same oscillatory errors previously identified in Fig. 4, which are particularly large for the mean streamwise velocity. In contrast, the REP-DRL control case shows a progressive reduction of the ${\bar{u}}^{+}$ error across training episodes, reaching a substantially smaller error of $\text{NRMSE}\;\left({\bar{u}}^{+}\right)\approx 3\cdot 10^{-2}$ by cumulative $t_{\text{avg}}^{+}=9.3\cdot 10^{3}$ (i.e., after 88 training episodes). However, the REP-DRL strategy exhibits a different trend for the streamwise velocity fluctuations; its error is maintained at a relatively constant level across the training process, with $\text{NRMSE}\;\left(u_{\text{rms}}^{+}\right)\approx 2\cdot 10^{-1}$. In contrast, the uncontrolled baseline error for $u_{\text{rms}}^{+}$ shows a downward trend, despite some oscillations, eventually converging toward approximately 10^−2^. Consequently, the performance gap widens throughout the training process, and the final REP-DRL error for this specific metric is an order of magnitude larger than the baseline value. Regarding the wall-normal velocity, Fig. 6(b) shows the errors of both its mean and fluctuating components remain nearly constant across the training process, with $\text{NRMSE}\left({\bar{v}}^{+}\right)∼10^{-5}$ and $\text{NRMSE}\left(v_{\text{rms}}^{+}\right)∼10^{-2}$. Finally, Fig. 6(c) indicates a clear convergence of the mean spanwise velocity, ${\bar{w}}^{+}$, until reaching its minimum error of $\text{NRMSE}\;\left({\bar{w}}^{+}\right)\approx 7\cdot 10^{-3}$ at a cumulative $t_{\text{avg}}^{+}\approx 2.2\cdot 10^{3}$ (corresponding to the end of training episode 17). Beyond this point, however, both the spanwise mean and fluctuating components begin to diverge from the optimal convergence path, exhibiting progressively larger errors as training advances.

**Table 3:** Comparison of velocity statistics NRMSE for the REP-DRL control and the uncontrolled baseline at key cumulative averaging times, $t_{\text{avg}}^{+}$, of the training process.

Control type	Cumulative $t_{\text{avg}}^{+}$	$t_{\text{avg}}^{+}$	NRMSE of velocity fields					
			${\bar{u}}^{+}$	${\bar{v}}^{+}$	${\bar{w}}^{+}$	$u_{\text{rms}}^{+}$	$v_{\text{rms}}^{+}$	$w_{\text{rms}}^{+}$
REP-DRL control	$2.2\cdot 10^{3}$	$6.0\cdot 10^{2}$	7.75e−2	4.91e−05	7.40e − 3	1.91e−1	9.81e−3	3.13e−2
Baseline, no control		$2.2\cdot 10^{3}$	4.97e−2	6.61e−06	6.85e − 3	9.27e−2	5.91e−3	1.18e−2

REP-DRL control	$9.3\cdot 10^{3}$	$6.0\cdot 10^{2}$	3.03e − 2	2.02e−05	6.41e−2	2.34e−1	1.00e−2	7.50e−2
Baseline, no control		$9.3\cdot 10^{3}$	1.32e − 1	4.38e−06	3.69e−3	1.77e−2	3.32e−3	6.77e−3

As corroborated by Table 3, this training process analysis provides valuable insight into the control problem, revealing a clear performance trade-off. This comparison highlights a critical distinction between the episodic training framework and the uninterrupted baseline. As detailed in the third column ($t_{\text{avg}}^{+}$) of Table 3, the REP-DRL statistics are evaluated over a single episodic window ($t_{\text{avg}}^{+}=6.0\cdot 10^{2}$), as the simulation is reset from an initially averaged restart file at the beginning of each episode as mentioned before. Conversely, the baseline statistics are integrated uninterruptedly across the equivalent cumulative duration of the REP-DRL training process, reaching cumulative $t_{\text{avg}}^{+}=2.2\cdot 10^{3}$ at episode 17 and $t_{\text{avg}}^{+}=9.3\cdot 10^{3}$ at episode 88. Consequently, the baseline benefits from a 3.6-fold and a 15.5-fold longer integration window than the REP-DRL agents at these respective checkpoints. This substantial difference in averaging time yields a statistical advantage for the baseline, as longer averaging windows naturally smooth out fluctuating fields. Therefore, the higher fluctuation errors observed for the REP-DRL case during training are primarily a consequence of incomplete statistical convergence within its short window, rather than a lack of physical control efficacy. In detail, while the ${\bar{u}}^{+}$ error continues to decrease after cumulative $t_{\text{avg}}^{+}=2.2\cdot 10^{3}$, ultimately reaching $\text{NRMSE}\;\left({\bar{u}}^{+}\right)\approx 3\cdot 10^{-2}$ at the end of the training, the spanwise ${\bar{w}}^{+}$ and $w_{\text{rms}}^{+}$ components begin to diverge precisely at cumulative $t_{\text{avg}}^{+}=2.2\cdot 10^{3}$. Meanwhile, the errors for $u_{\text{rms}}^{+}$, ${\bar{v}}^{+}$, and $v_{\text{rms}}^{+}$ remain approximately constant throughout the entire training process.

The network weights from training episode 17, corresponding to cumulative $t_{\text{avg}}^{+}=2.2\cdot 10^{3}$, are selected for subsequent unsupervised deployment studies in Sections 5.3, 5.4, and 5.5. Within this single supervised training trajectory, the network weights from episode 17 provide the most balanced compromise among the targeted velocity statistics, achieving significant convergence acceleration in the mean spanwise velocity (${\bar{w}}^{+}$) prior to the onset of divergence, while preserving moderate gains in ${\bar{u}}^{+}$ and maintaining numerical stability across remaining velocity statistics. These trained network weights are saved and used for the unsupervised performance and sensitivity analyses presented in the following subsections. Therefore, the control policy selection is based exclusively on supervised training diagnostics and does not incorporate any information from subsequent evaluation runs.

**Fig. 7:**
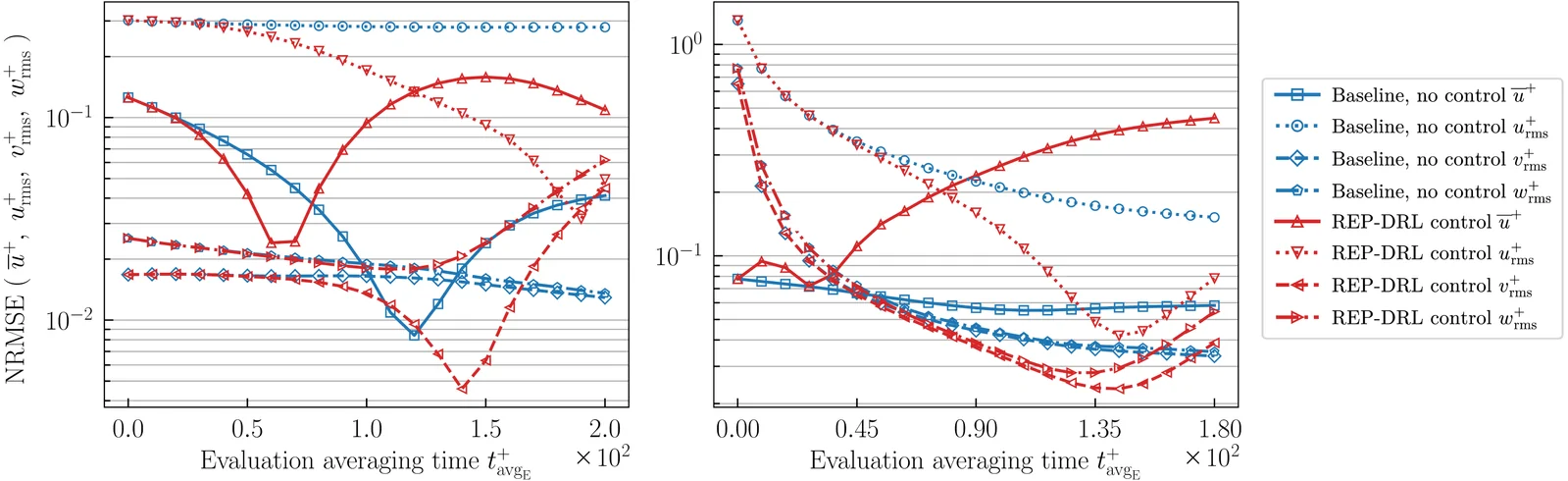
NRMSE temporal evolution for (left) Case I at $Re_{\tau }=100$, and (right) Case II at $Re_{\tau }=180$. The error history is represented with respect to the evaluation averaging time $t_{{\text{avg}}_{\text{E}}}^{+}$. Blue lines denote the uncontrolled baseline and red lines represent the REP-DRL-controlled case. (For interpretation of the references to color in this figure legend, the reader is referred to the web version of this article.)

### 5.3 Performance analysis

The performance of the optimized REP-DRL control policy is evaluated first on the training configuration, Case I ($Re_{\tau }=100$), using a distinct restart file to ensure robustness across different initial flow fields. To further assess the method’s generalization and extrapolation capabilities, a more challenging evaluation is conducted on Case II ($Re_{\tau }=180$), representing a significantly more turbulent environment. The selected policy checkpoint at training episode 17 is fixed prior to any evaluation (see Section 5.2), and remains unchanged throughout all subsequent unsupervised performance evaluations in both Case I and Case II. In both evaluation scenarios, the unsupervised approach is employed; viz., the observation [Eq. (26)] and reward [Eq. (32)] error-based expressions use flow state from the previous control step as the reference state, i.e., $t_{r}=t_{m-1}$ as defined in Eq. (24). All results are analyzed in terms of the evaluation averaging time $t_{{\text{avg}}_{\text{E}}}^{+}=t_{\text{avg}}^{+}-t_{{\text{avg}}_{0}}^{+}$, with $t_{{\text{avg}}_{0}}^{+}$ detailed in Table 2. As specified in Section 5.1 for all performance evaluations presented in this section, this performance analysis is strictly bounded to the active control window ($t_{\text{eval}}^{+}$) under active body forcing ($f_{\text{REP}}\neq 0$). As noted in Section 3.5, because post-actuation continuation simulations under $f_{\text{REP}}=0$ are not conducted in this study, the performance trends presented herein demonstrate convergence acceleration during active forcing to achieve target statistics tolerances at an specific time instance rather than the permanent adoption of an accelerated trajectory by the unforced dynamics. Therefore, upon force deactivation, the physical flow is expected to undergo transient relaxation back toward the unforced baseline dynamics.

The quantitative impact of the REP-DRL control on statistics convergence is presented in Fig. 7, which compares the NRMSE history of the REP-DRL-controlled simulation (red) against the uncontrolled baseline (blue). To contextualize these trends relative to the training phase presented in Section 5.2 (specifically Table 3 and Fig. 6), it is important to note that the evaluation protocol eliminates the averaging window imbalance. During training, the uninterrupted baseline simulation benefited from a significantly longer integration window than the selected REP-DRL training episodes (3.6-fold and 15.5-fold longer at the respective checkpoints in Table 3), which naturally reduced baseline fluctuation errors due to time-averaging smoothing. In contrast, during this evaluation stage, the uncontrolled baseline and the REP-DRL controlled case are evaluated under strictly identical conditions: both cases are initiated from the same pre-averaged restart file ($t_{{\text{avg}}_{0}}^{+}$) and accumulate averaging time concurrently over the same evaluation window duration, $t_{{\text{avg}}_{\text{E}}}^{+}$. Under this evaluation framework, both simulations compute global NRMSE values relative to the same high-fidelity converged reference state ($t_{{\text{avg}}_{\text{C}}}^{+}\gg t_{{\text{avg}}_{\text{E}}}^{+}$) whereas the REP-DRL control agents operate in an entirely unsupervised manner using $t_{r}=t_{m-1}$ for local state and reward updates. By enforcing equal averaging window lengths, the baseline’s statistical averaging advantage is eliminated, thereby demonstrating the true physical capability of the learned REP-DRL policy to accelerate the convergence of the velocity statistics.

For Case I, the analysis of the left sub-figure reveals a significant convergence acceleration of the velocity fluctuations. This is particularly evident in the mean streamwise velocity fluctuations, where the $u_{\text{rms}}^{+}$ error reaches its minimum of $\text{NRMSE}\left(u_{\text{rms}}^{+}\right)\approx 3\cdot 10^{-2}$ at $t_{{\text{avg}}_{\text{E}}}^{+}=1.9\cdot 10^{2}$, while the baseline error remains high at $\text{NRMSE}\left(u_{\text{rms}}^{+}\right)>2\cdot 10^{-1}$ over the whole evaluation period. The wall-normal velocity fluctuations ($v_{\text{rms}}^{+}$) also shows rapid convergence for the REP-DRL-controlled case, reaching $\text{NRMSE}\left(v_{\text{rms}}^{+}\right)\approx 4\cdot 10^{-3}$ at $t_{{\text{avg}}_{\text{E}}}^{+}\approx 1.4\cdot 10^{2}$, a value significantly lower than the baseline minimum of $\text{NRMSE}\left(v_{\text{rms}}^{+}\right)\approx 2\cdot 10^{-2}$. The spanwise velocity fluctuations ($w_{\text{rms}}^{+}$) for the REP-DRL-controlled flow show similar behavior to the uncontrolled baseline up until $t_{{\text{avg}}_{\text{E}}}^{+}\approx 1.4\cdot 10^{2}$. However, after $t_{{\text{avg}}_{\text{E}}}^{+}\approx 1.4\cdot 10^{2}$, representing the 140% of the training episode duration, the convergence of both the controlled $v_{\text{rms}}^{+}$ and $w_{\text{rms}}^{+}$ begin to degenerate. Furthermore, the persistent, large-amplitude oscillations observable for the streamwise velocity still impact the convergence of the mean streamwise velocity (${\bar{u}}^{+}$) for both the uncontrolled baseline and the REP-DRL controlled simulations, which show similar oscillatory patterns in the ${\bar{u}}^{+}$ error evolution. This behavior is a known artifact of low-frequency oscillations associated with streamwise coherent structures that are permitted by the periodic boundary conditions in the streamwise and spanwise directions. In more applied scenarios with inflow/outflow boundaries, these specific low-frequency errors are not expected to be a dominant factor, suggesting the policy’s effectiveness on the mean flow, and corresponding fluctuations, could be even greater.

For Case II, the right sub-figure evaluates the agents’ adaptability to a more turbulent flow case. The REP-DRL control strategy successfully accelerates the initial convergence rate for all velocity fluctuation components up to $t_{{\text{avg}}_{\text{E}}}^{+}\approx 1.35\cdot 10^{2}$, reaching smaller error values than the uncontrolled baseline. In particular, the streamwise fluctuations ($u_{\text{rms}}^{+}$) show a striking convergence improvement, reaching $\text{NRMSE}\left(u_{\text{rms}}^{+}\right)\approx 4\cdot 10^{-2}$ in contrast to the approximately $2\cdot 10^{-1}$ of the uncontrolled baseline at that evaluation averaging time, $t_{{\text{avg}}_{\text{E}}}^{+}\approx 1.35\cdot 10^{2}$. However, the control policy, which has not been trained in this flow regime, induces a progressive decrease in the bulk streamwise velocity. This manifests as a monotonic error growth in the mean streamwise velocity (${\bar{u}}^{+}$) starting from $t_{{\text{avg}}_{\text{E}}}^{+}\approx 0.45\cdot 10^{2}$, which subsequently contaminates the estimates of the fluctuating velocity components.

This behavior provides a clear physical confirmation of the unsupervised policy drift hypothesized in Section 4.3.4, exposing the intrinsic non-linear coupling that binds the mean flow profile to the Reynolds stress tensor. Physically, the mean streamwise velocity and the turbulent fluctuations are dynamically coupled through the production of turbulent kinetic energy via the Reynolds shear stress and the mean shear gradient ($P_{k}=-\bar{u^{\prime }v^{\prime }}\partial \bar{u}/\partial y$). Consequently, while the volumetric forcing $f_{\text{REP}}$ introduced by the DRL agent successfully manipulates the Reynolds stress eigenspace to accelerate fluctuation convergence, it inevitably alters this local production mechanism. Additionally, as the policy is deployed in an unseen, higher-Reynolds-number regime ($Re_{\tau }=180$) where no high-fidelity converged flow statistics are available for the state [Eq. (26)] and reward [Eq. (32)] evaluations (using $t_{r}=t_{m-1}$), the resulting perturbation actions inadvertently alter the mean streamwise velocity profile. While this statistics drift reveals a specific vulnerability of the unsupervised REP-DRL approach when applied to the higher-Reynolds-number extrapolation case, the framework still provides a remarkable initial acceleration of the velocity fluctuations convergence without requiring any high-fidelity reference data.

**Fig. 8:**
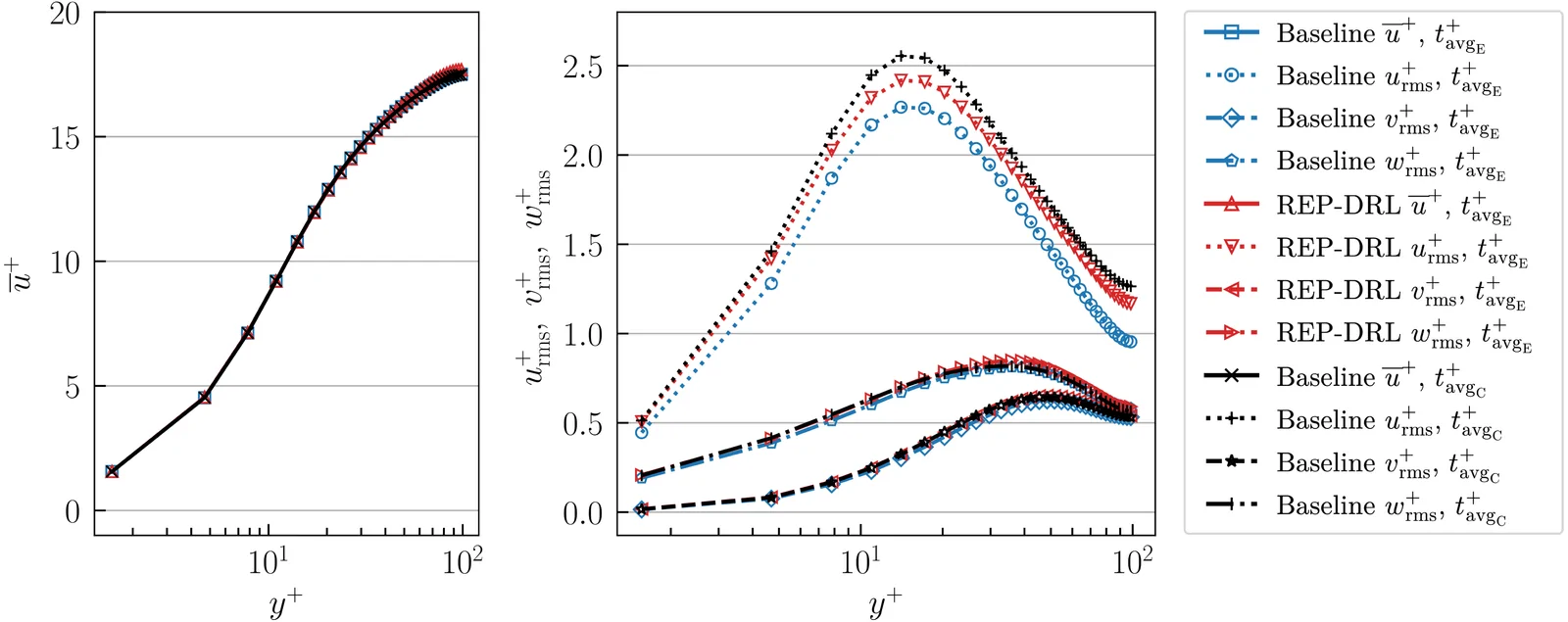
Mean and fluctuating velocity profiles for Case I ($Re_{\tau }=100$). The REP-DRL-controlled case (red) and non-converged, uncontrolled baseline (blue) at $t_{{\text{avg}}_{\text{E}}}^{+}=1.5\cdot 10^{2}$ are compared against the final converged reference at $t_{{\text{avg}}_{\text{C}}}^{+}=4\cdot 10^{4}$ (black). (For interpretation of the references to color in this figure legend, the reader is referred to the web version of this article.)

It should be emphasized that the evaluation window $t_{{\text{avg}}_{\text{E}}}^{+}=1.35\cdot 10^{2}$ utilized for Case II analysis is determined offline by comparing the controlled flow against a fully converged DNS reference to establish the lower bound of target statistics error and demonstrate initial acceleration capability. Nevertheless, in a practical, truly unsupervised deployment where ground-truth reference statistics are unavailable, simulation termination cannot rely on offline error thresholds. Instead, autonomous deployment termination can be governed by data-driven stopping criteria, such as monitoring statistics trajectory stability via the Cauchy criterion ($\left|\phi_{n}-\phi_{m}\right|<\epsilon$ for n,m>K) (Rudin, 1976), or systematically tracking statistics stationarity and finite-sampling uncertainties running batch-means or autocorrelation models (Oliver et al., 2014, Russo and Luchini, 2017).

Nevertheless, this result is noteworthy, as it demonstrates that the REP-DRL learned control policy remains physically relevant even in an unseen, more turbulent flow, providing a significant initial acceleration in the convergence of the velocity fluctuations. Additionally, the comparison between the unsupervised deployments of Case I ($Re_{\tau }=100$) and Case II ($Re_{\tau }=180$) indicates that, while the learning policy continues to exhibit effective performance in accelerating the initial convergence of the velocity fluctuation components across both Reynolds numbers, its long-term behavior shows increased sensitivity to the flow regime. In particular, the stronger deviations observed in the mean streamwise velocity for Case II suggest that the policy does not fully transfer across different Reynolds-number regimes, with performance progressively degrading as the flow departs from the training conditions due to modifications in the underlying turbulent dynamics. While the control policy is therefore not stable for long-term integration in the higher-Reynolds-number regime, its initial success in Case II strongly suggests that training on a curriculum of Reynolds numbers is a critical next step toward achieving robust and stable generalization strategy.

**Fig. 9:**
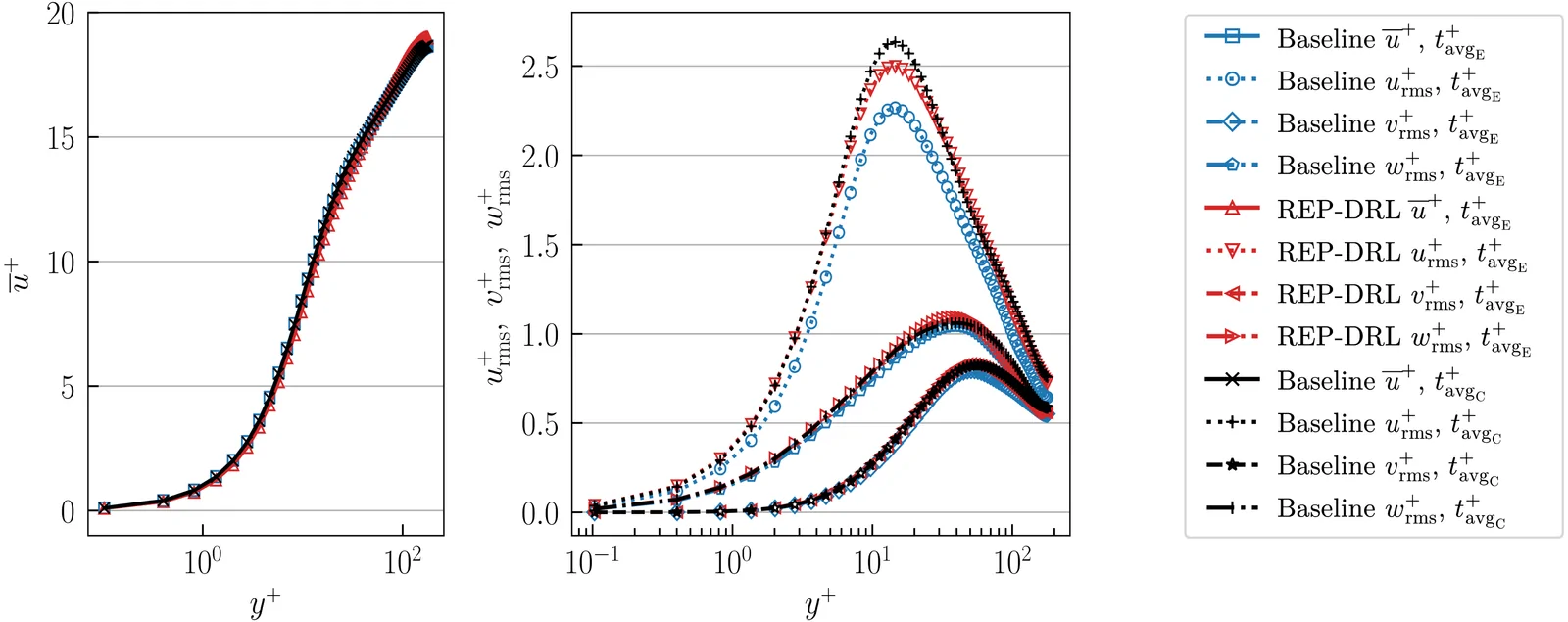
Mean and fluctuating velocity profiles for Case II ($Re_{\tau }=180$). The REP-DRL-controlled case (red) and non-converged, uncontrolled baseline (blue) at $t_{{\text{avg}}_{\text{E}}}^{+}=1.35\cdot 10^{2}$ are compared against the final converged reference at $t_{{\text{avg}}_{\text{C}}}^{+}∼3\cdot 10^{4}$ (black). (For interpretation of the references to color in this figure legend, the reader is referred to the web version of this article.)

To connect these global error metrics to the underlying physics, the velocity profiles are examined at specific evaluation times corresponding to 30 perturbation actions, in contrast to the $t_{\text{ep}}^{+}/t_{\text{act}}^{+}=20$ perturbation actions per training episode. This correspond to evaluation averaging times of $t_{{\text{avg}}_{\text{E}}}^{+}=1.5\cdot 10^{2}$ for Case I and $t_{{\text{avg}}_{\text{E}}}^{+}=1.35\cdot 10^{2}$ for Case II. Fig. 8, Fig. 9 compare the targeted statistics profiles from the REP-DRL-controlled simulations (red) against both the non-converged, uncontrolled baseline at the same averaging time (blue) and the final converged reference (black). In both evaluated cases, the REP-DRL-controlled flow achieves velocity profiles that show better agreement with the final converged solution. This is especially significant for the velocity fluctuation components ($u_{\text{rms}}^{+},v_{\text{rms}}^{+},w_{\text{rms}}^{+}$), demonstrating that the perturbing agents rapidly guide the simulation toward the converged statistical state.

A more fundamental assessment of the turbulent state is provided by the Reynolds stresses anisotropy, illustrated in Fig. 10 using barycentric maps. These representations visualize the nature of turbulence based on the methodology detailed in subsection 3.3. For Case I, Fig. 10(a) shows that the non-converged baseline data (blue) is scattered and undeveloped, far from the characteristic *C* shape of the converged reference solution (black). Conversely, the data from the REP-DRL-controlled flow (red) already traces the converged profile with only a small bias. For Case II, Fig. 10(b) reveals a particularly compelling result: the REP-DRL-controlled flow data (red) follows the reference shape almost perfectly, in contrast with the non-converged uncontrolled baseline (blue), which exhibits significant deviations in the interior of the channel. This advanced analysis confirms that the REP-DRL control agents accelerate not just the convergence of first- and second-order statistical moments but also the rapid development of the correct underlying turbulent structure.

Fig. 10Barycentric map of $R_{ij}$ anisotropy for (a) Case I at $Re_{\tau }=100$, and (b) Case II at $Re_{\tau }=180$. Non-converged, uncontrolled baseline (blue squares) and REP-DRL-controlled case (red triangles) are shown at $t_{{\text{avg}}_{\text{E}}}^{+}$; converged reference at $t_{{\text{avg}}_{\text{C}}}^{+}$ is shown as black crosses. All markers are shaded by the relative wall-normal position y/δ. Averaging times are $t_{{\text{avg}}_{\text{E}}}^{+}=1.5\cdot 10^{2}$ and $t_{{\text{avg}}_{\text{C}}}^{+}=4\cdot 10^{4}$ for (a); $t_{{\text{avg}}_{\text{E}}}^{+}=1.35\cdot 10^{2}$ and $t_{{\text{avg}}_{\text{C}}}^{+}∼3\cdot 10^{4}$ for (b).Fig. 10

**(a):**
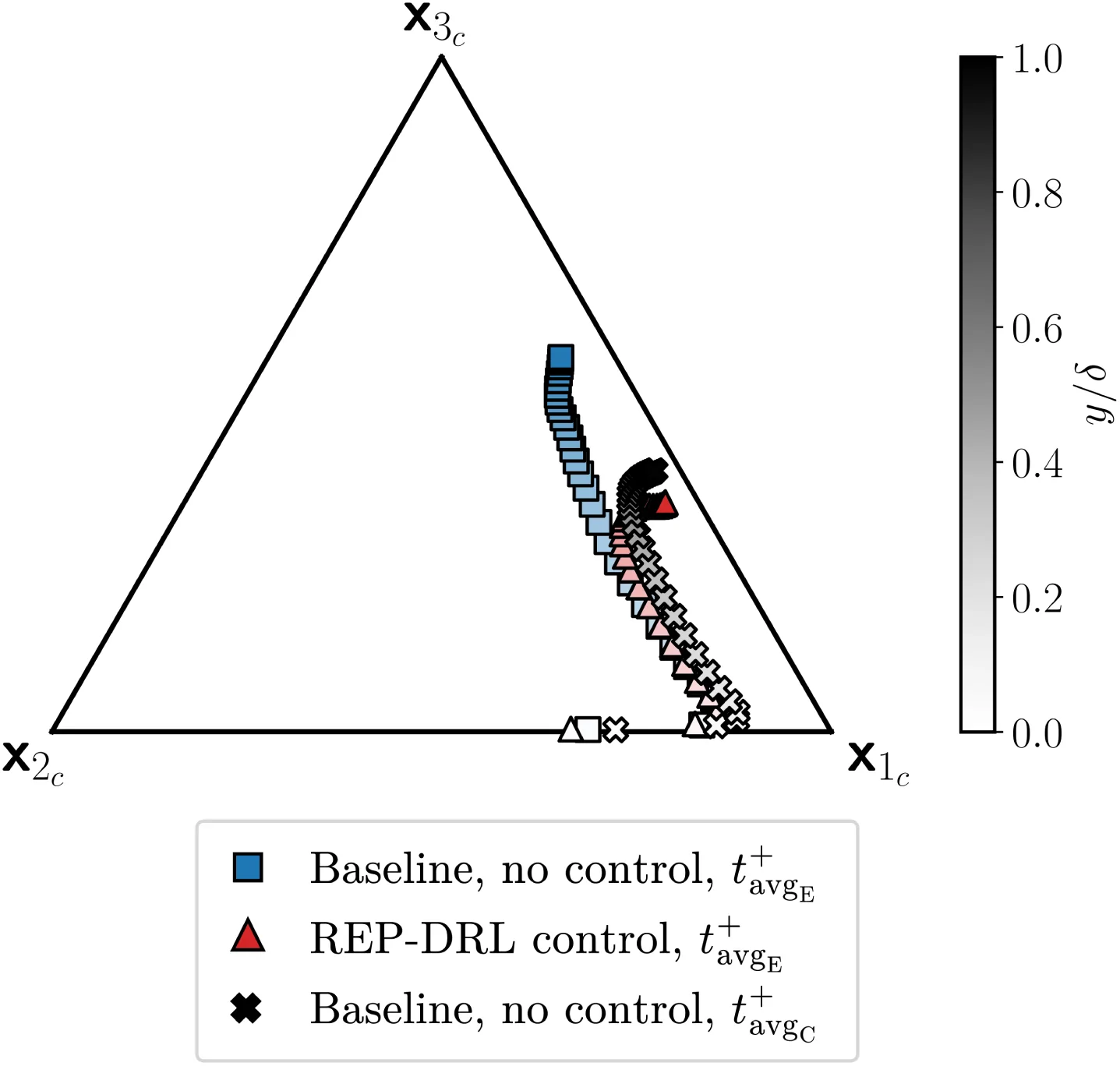
.

**(b):**
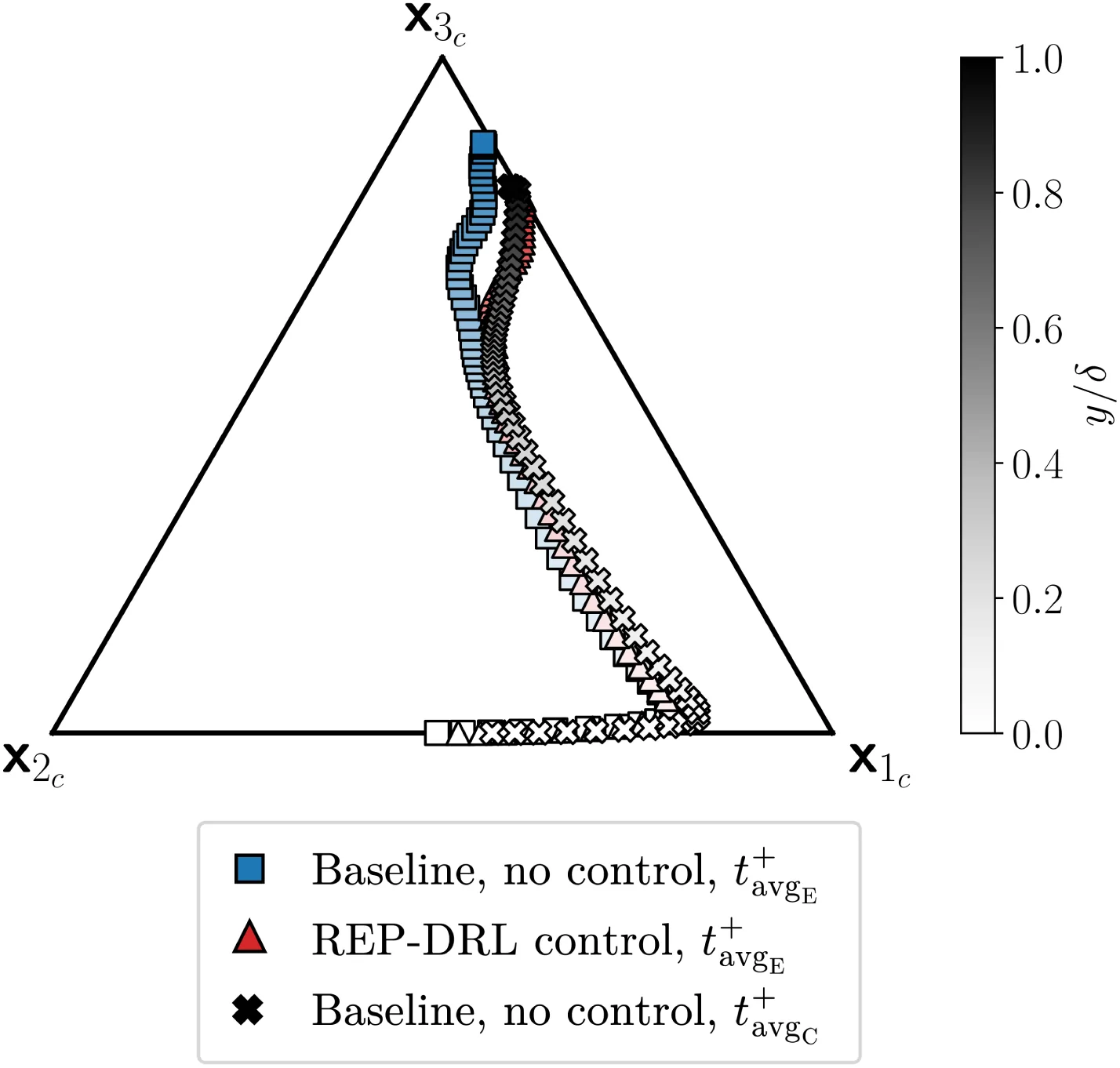
.

### 5.4 Sensitivity to actuation period

**Fig. 11:**
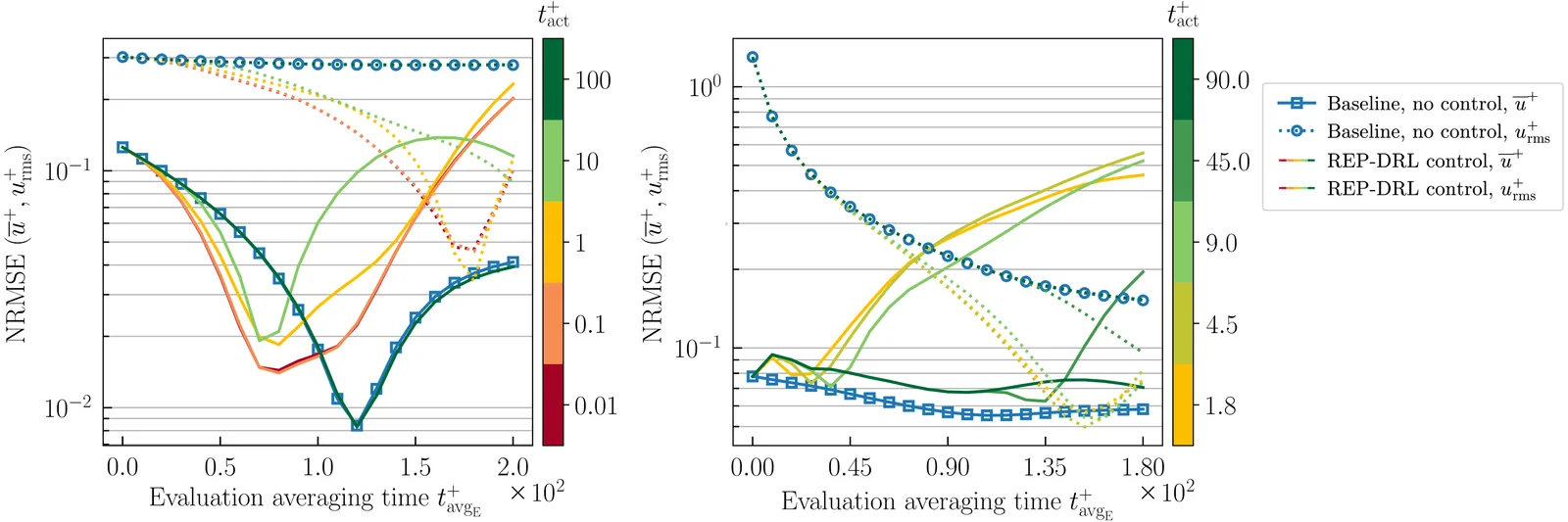
Sensitivity analysis of the REP-DRL control to the non-dimensional actuation period, $t_{\text{act}}^{+}=t_{\text{act}}/t_{\tau }$. The NRMSE of streamwise velocity statistics (${\bar{u}}^{+}$ and $u_{\text{rms}}^{+}$) is shown as a function of the evaluation averaging time $t_{{\text{avg}}_{\text{E}}}^{+}$ for (left) Case I at $Re_{\tau }=100$, and (right) Case II at $Re_{\tau }=180$.

A sensitivity analysis is performed to evaluate the effect of the actuation period, $t_{\text{act}}$, on the unsupervised performance of the REP-DRL trained policy. The control framework, trained using $t_{\text{act}}^{+}=t_{\text{act}}/t_{\tau }=5$ as detailed in Table 2, is re-evaluated using a broad range of actuation period values: $t_{\text{act}}^{+}=\left\{0.01,0.1,1,10,100\right\}$ for Case I, and $t_{\text{act}}^{+}=\left\{1.8,4.5,9,45,90\right\}$ for Case II. The results for the streamwise velocity statistics are presented in Fig. 11.

For Case I ($Re_{\tau }=100$), the left sub-figure reveals a strong and non-monotonic dependency on the actuation period. At one extreme, the slowest actuation period of $t_{\text{act}}^{+}=100$ (dark green) is ineffective, almost matching the uncontrolled baseline (blue) and rendering the REP-DRL control negligible. At the other extreme, high actuation frequency ($t_{\text{act}}^{+}\leq 1$) proves to be unstable for the mean streamwise velocity (${\bar{u}}^{+}$) convergence. For instance, $t_{\text{act}}^{+}=1$ (yellow) shows a rapid initial decrease in $u_{\text{rms}}^{+}$ error, reaching $\text{NRMSE}\left(u_{\text{rms}}^{+}\right)\approx 6\cdot 10^{-2}$ at $t_{{\text{avg}}_{\text{E}}}^{+}\approx 1.75\cdot 10^{2}$, but its corresponding ${\bar{u}}^{+}$ error becomes unstable and diverges by the end of the evaluation time. The most effective and stable control strategy is achieved by $t_{\text{act}}^{+}=10$ (light green), which maintains a stable, oscillating error for ${\bar{u}}^{+}$ (similar to the uncontrolled baseline and caused by inherent low-frequency flow oscillations), while providing significant and monotonic convergence for the fluctuating component ($u_{\text{rms}}^{+}$). In particular, this REP-DRL control frequency achieves a final $\text{NRMSE}\left(u_{\text{rms}}^{+}\right)\approx 0.1$ at $t_{{\text{avg}}_{\text{E}}}^{+}=2\cdot 10^{2}$, comparable to higher actuation frequencies but maintaining a stable ${\bar{u}}^{+}$ convergence.

In contrast, the sensitivity analysis for Case II ($Re_{\tau }=180$), presented in the right sub-figure of Fig. 11, demonstrates the policy’s complex generalization behavior, revealing a significant trade-off while achieving highly promising results for $u_{\text{rms}}^{+}$ convergence. Stability of the mean streamwise velocity (${\bar{u}}^{+}$) is only achieved at the slowest, ineffective actuation frequencies (corresponding to $t_{\text{act}}^{+}=45$ and 90), which largely replicate the baseline behavior. In contrast, all faster, active control frequencies (corresponding to $t_{\text{act}}^{+}=1.8,\;4.5$, and 9) result in an immediate divergence of ${\bar{u}}^{+}$. In a compelling demonstration of the method’s potential, however, these same control frequencies provide remarkable initial acceleration for the streamwise velocity fluctuations (${\bar{u}}^{+}$). Specifically, similar $t_{\text{act}}^{+}=\left\{1.8,\;4.5,\;9\right\}$ drive the $\text{NRMSE}\left(u_{\text{rms}}^{+}\right)$ down to a minimum of approximately $6\cdot 10^{-2}$ around $t_{{\text{avg}}_{\text{E}}}^{+}\approx 1.35\cdot 10^{2}$.

In summary, this analysis confirms the framework’s potential, revealing that its performance is a complex interplay between control frequency and the target statistic. For Case I, a moderate actuation period of $t_{\text{act}}^{+}=10$ appears optimal, balancing ${\bar{u}}^{+}$ stability and $u_{\text{rms}}^{+}$ convergence acceleration, while faster frequencies proved unstable. For Case II, the analysis provides a key insight: the very control frequencies that destabilize the mean flow are precisely those that are most effective for accelerating the fluctuations. This discovery, rather than being a limitation, highlights a rich area for future work and strongly suggests that a more advanced (e.g., multi-objective) agent could learn to harness this complex behavior, potentially leading to control strategies that optimize all mean and fluctuating velocity components simultaneously.

### 5.5 Net computational cost and amortization analysis

To evaluate the practical viability of the proposed REP-DRL framework, a comprehensive computational cost analysis is performed. As established at the beginning of Section 5.2, the overall computational expense of the REP-DRL framework is governed almost entirely by the underlying numerical flow solver, rendering the overhead introduced by the DRL operations (state sensing, reward calculation, network updates, and $R_{ij}$ eigen-decomposition) negligible. Consequently, the computational cost C of any simulation run, including training, deployment, or baseline evaluation, scales linearly with the total number of advanced temporal integration steps, $N_{t}$, across training, deployment and baseline evaluations.

The total computational cost of the proposed framework accounts for the one-time training overhead ($C_{\text{train}}$) and the deployment cost ($C_{\text{deploy}}$) accumulated over M independent evaluation runs. A net computational saving is achieved if the combined cost of the REP-DRL training and deployment is strictly lower than the cost of the uncontrolled baseline ($C_{\text{base}}$) required to reach identical error thresholds ($\epsilon_{1}$, $\epsilon_{2}$, and $\epsilon_{3}$) for the fluctuating velocity components. This condition is expressed as

(33) $$C_{\text{train}}+MC_{\text{deploy}}<MC_{\text{base}}.$$

From Eq. (33), the minimum number of independent evaluation runs, $M_{\text{min}}$, required to fully amortize the initial training investment is defined by

(34) $$M>M_{\text{min}}=\frac{C_{\text{train}}}{C_{\text{base}}-C_{\text{deploy}}}.$$

To calculate this threshold accurately, computational costs are evaluated under two distinct temporal discretization paradigms: a conservative fixed time step (denoted as Δt or $\Delta t_{c}^{+}$ in Table 1) for numerical stability, and an optimal time step ($\Delta t_{\text{op}}^{+}$ in Table 1) derived from the Courant–Friedrichs–Lewy (CFL) stability condition (Ferziger et al., 2002) with CFL=0.5. Additionally, the training overhead is calculated for both the complete exploratory training process ($N_{\text{ep}}=100$, see Table 2) and an idealized early-stopped training ($N_{\text{ep}}=17$) corresponding to the identification of the optimal control policy and subsequent ending of the training process.

**Table 4:** End-to-end computational cost in terms of $N_{t}$ advanced time steps and computational cost amortization threshold $M_{\text{min}}$ individual deployment simulations of the REP-DRL framework, trained at $Re_{\tau }=100$ and evaluated at $Re_{\tau }=180$ for the convergence acceleration of the velocity fluctuating components.

Training process ($N_{\text{ep}}$)	Time step ($\Delta t^{+}$)	$C_{\text{train}}$ ($N_{t}$)	$C_{\text{deploy}}$ ($N_{t}$)	$C_{\text{base}}$ ($N_{t}$)	Amortization ($M_{\text{min}}$)
Exploratory (100 Ep.)	Conservative ($\Delta t_{c}^{+}$)	$1.0\cdot 10^{6}$	$3.5\cdot 10^{4}$	$1.4\cdot 10^{5}$	M≥10
Exploratory (100 Ep.)	CFL-Optimal ($\Delta t_{\text{op}}^{+}$)	$1.8\cdot 10^{4}$	$1.4\cdot 10^{3}$	$5.4\cdot 10^{3}$	M≥5
Early-Stopped (17 Ep.)	Conservative ($\Delta t_{c}^{+}$)	$2.2\cdot 10^{5}$	$3.5\cdot 10^{4}$	$1.4\cdot 10^{5}$	M≥3
Early-Stopped (17 Ep.)	CFL-Optimal ($\Delta t_{\text{op}}^{+}$)	$4.1\cdot 10^{3}$	$1.4\cdot 10^{3}$	$5.4\cdot 10^{3}$	M≥2

It is important to note that while all baseline, training, and deployment simulations in this work have been executed using the conservative time step ($\Delta t_{c}^{+}$), the CFL-optimal results (evaluated using $\Delta t_{\text{op}}^{+}$) reported in Table 4 represent projected lower bounds on the amortization threshold. These projected values are constructed by scaling the DNS execution cost ($N_{t}$) according to the ratio of temporal steps ($\Delta t_{c}^{+}/\Delta t_{\text{op}}^{+}$), while holding the total physical duration ($t_{\text{ep}}^{+},t_{\text{eval}}^{+}$) and control actuation period ($t_{\text{act}}^{+}$) strictly constant. This direct analytical projection is justified by three key considerations. First, the control actuation period $t_{\text{act}}^{+}$ is an explicit physical timescale of the control framework, defined independently of the DNS numerical integration step $\Delta t^{+}$. Therefore, whether the flow is advanced between control steps using fine integration sub-steps ($N_{c}=t_{\text{act}}^{+}/\Delta t_{c}^{+}$) or fewer optimal sub-steps ($N_{\text{op}}=t_{\text{act}}^{+}/\Delta t_{\text{op}}^{+}<N_{c}$), the control policy executes updates at identical physical time intervals. Second, because both $\Delta t_{c}^{+}$ and $\Delta t_{\text{op}}^{+}$ lie within the temporal accuracy and numerical stability limits of the solver (CFL≤0.5), time-stepping integration errors remain minimal across both discretizations, ensuring that the resolved turbulent dynamics and low-frequency statistical oscillations (Section 4.2) remain physically identical. Third, because the DRL control policy operates on macroscopic, time-integrated running statistics (velocity-error-based state [Eq. (26)] and reward [Eq. (32)]) accumulated over macroscopic windows ($t_{\text{act}}^{+}\gg \Delta t_{c}^{+},\;\Delta t_{\text{op}}^{+}$), the environment state inputs, reward signals, and resulting policy action outputs are invariant to DNS time step adjustments between $\Delta t_{c}^{+}$ and $\Delta t_{\text{op}}^{+}$, allowing learned control policies to transfer directly without modification.

The computational costs associated with the training and deployment of the REP-DRL framework, alongside the cost of the uncontrolled baseline simulation, are detailed below.

• Training cost ($C_{\text{train}}$): Full REP-DRL exploratory training ($N_{\text{ep}}=100$) accumulates a total simulation time of $t_{\text{train,total}}^{+}=10^{4}$. This requires $N_{\text{t,c}}=10^{6}$ integration steps under the conservative configuration ($\Delta t_{\text{c}}^{+}=10^{-2}$ for Case I at $Re_{\tau }=100$), or $N_{\text{t,op}}=1.8\cdot 10^{4}$ steps using the optimal time step ($\Delta t_{\text{op}}^{+}=5.4\cdot 10^{-1}$ for Case I at $Re_{\tau }=100$). Alternatively, early-stopping at the optimal training checkpoint (episode 17, at cumulative averaging time $t_{\text{train,17}}^{+}=2.2\cdot 10^{3}$) yields a significant lower training cost with $N_{\text{t,c,17}}=2.2\cdot 10^{5}$ steps using the conservative time step ($\Delta t_{\text{c}}^{+}$), or $N_{\text{t,op,17}}=4.1\cdot 10^{3}$ steps for the optimal time step ($\Delta t_{\text{op}}^{+}$).
• Deployment cost ($C_{\text{deploy}}$): During the unsupervised evaluation phase at $Re_{\tau }=180$ (Case II), the optimized control policy aims to accelerate the convergence of turbulent statistics. In this proof-of-concept benchmark, $C_{\text{deploy}}$ is evaluated over a minimum averaging window of $t_{{\text{avg}}_{\text{E}}}^{+}=1.35\cdot 10^{2}$ identified offline using the converged reference state, corresponding to the peak acceleration window where fluctuating velocity components reach targeted error thresholds (see right panel of Fig. 7). Including the pre-averaging window of the restart file, the total time advancement is $t_{\text{avg}}^{+}=t_{{\text{avg}}_{0}}^{+}+t_{{\text{avg}}_{\text{E}}}^{+}=3.15\cdot 10^{2}$, requiring $N_{\text{t,c}}=3.5\cdot 10^{4}$ conservative steps ($\Delta t_{\text{c}}^{+}=9\cdot 10^{-3}$ for Case II, see Table 1), or $N_{\text{t,op}}=1.4\cdot 10^{3}$ optimal steps ($\Delta t_{\text{op}}^{+}=2.3\cdot 10^{-1}$ for Case II). At this stage of the averaging process, the fluctuating velocity components have reached normalized root-mean-squared errors (NRMSE) of $\epsilon_{1}=\text{NRMSE}\left(u_{\text{rms}}\right)=5\cdot 10^{-2}$, $\epsilon_{2}=\text{NRMSE}\left(v_{\text{rms}}\right)=2.5\cdot 10^{-2}$, and $\epsilon_{3}=\text{NRMSE}\left(w_{\text{rms}}\right)=3\cdot 10^{-2}$ (see right panel of Fig. 7).
• Baseline cost ($C_{\text{base}}$): As illustrated by the uncontrolled baseline convergence trajectories in the right panel of Fig. 4, reaching equivalent NRMSE for the velocity fluctuating components ($\epsilon_{1}$, $\epsilon_{2}$, and $\epsilon_{3}$) requires a significantly longer integration window of $t_{\text{avg}}^{+}=1.25\cdot 10^{3}$ to reach all targeted $\epsilon_{1}$, $\epsilon_{2}$ and $\epsilon_{3}$. This temporal advancement corresponds to $N_{\text{t,c}}=1.4\cdot 10^{5}$ conservative steps ($\Delta t_{\text{c}}^{+}=9\cdot 10^{-3}$ for Case II) or $N_{\text{t,op}}=5.4\cdot 10^{3}$ optimal steps ($\Delta t_{\text{op}}^{+}=2.3\cdot 10^{-1}$ for Case II).

The quantitative amortization thresholds ($M_{\text{min}}$) derived from Eq. (34) are compiled in Table 4. To clearly interpret these results, each row of Table 4 is detailed below, representing a specific operational configuration with a distinct amortization threshold.

• Exploratory training with conservative time step (M≥10): Represents the baseline experimental framework executed in this work, where the agent undergoes full 100-episode exploration under $\Delta t_{c}^{+}$. In this scenario, 10 independent deployment runs are required to fully recover the initial training investment.
• Exploratory training with CFL-optimal time step (M≥5): Represents the projected scenario of optimizing the numerical integration step to $\Delta t_{\text{op}}^{+}$ (CFL=0.5) while maintaining full exploratory training. This numerical optimization cuts the amortization threshold in half ($M_{\text{min}}=5$) relative to the executed baseline.
• Early-stopped training with conservative time step (M≥3): Highlights the idealized computational savings achieved if training is terminated once the policy reaches peak performance at episode 17, reducing the amortization threshold to 3 deployment runs under the executed conservative time step $\Delta t_{c}^{+}$.
• Early-stopped training with CFL-optimal time step (M≥2): Represents the idealized lower bound on the amortization threshold ($M_{\text{min}}=2$), which combines the early, retrospective selection of episode 17 as the optimal policy checkpoint and the use of the CFL-optimal time step ($\Delta t_{\text{op}}^{+}$).

Overall, depending on the training duration and early-stopping criteria, the initial training overhead is recovered within 3 to 10 deployment runs under the executed conservative time steps, and within 2 to 5 runs under projected CFL-optimal conditions.

Crucially, the REP-DRL deployment costs ($C_{\text{deploy}}$) and the corresponding amortization thresholds ($M_{\text{min}}$) reported in Table 4 are computed using the single policy checkpoint (episode 17) derived from the primary training run. In this context, the resulting $M_{\text{min}}$ thresholds serve as a proof-of-concept benchmark, demonstrating that the REP-DRL framework can achieve substantial per-deployment savings ($\Delta C=C_{\text{base}}-C_{\text{deploy}}$) relative to the initial training investment. While stochastic variance across independent training seeds may alter exact training efficiency and deployment performance, with an exhaustive multi-seed ensemble study left for future work as discussed in Section 6, the low order of magnitude of $M_{\text{min}}$ values highlights the physical efficiency of the proposed REP-DRL methodology.

To contextualize these amortization metrics, the validity and operational scope of this computational cost analysis are bounded by six key physical and computational assumptions. First, the computational cost analysis is strictly valid for the convergence acceleration of the velocity fluctuation components ($u_{\text{rms}}^{+}$, $v_{\text{rms}}^{+}$, $w_{\text{rms}}^{+}$). It is important to highlight that while the ${\bar{u}}^{+}$ error carries a significant weight in the reward function [Eq. (32)] to act as a physical regularizer against mean momentum balance distortions caused by stress perturbations, its convergence trajectory experiences performance degradation under the distinct extrapolation conditions of Case II ($Re_{\tau }=180$) when trained solely on Case I ($Re_{\tau }=100$). Specifically, as shown in Fig. 7 (right), ${\bar{u}}^{+}$ exhibits a minor long-term drift that prevents it from persistently remaining bounded within the target error threshold over extended deployments. Consequently, the cost-saving claims in this subsection are conservatively restricted to the second-order velocity fluctuations ($u_{\text{rms}}^{+}$, $v_{\text{rms}}^{+}$, $w_{\text{rms}}^{+}$), which represent the primary computational bottleneck in DNS statistics convergence and strictly satisfy target bounds up to the deployment time window $t_{{\text{avg}}_{\text{E}}}^{+}\approx 1.35\cdot 10^{2}$ for Case II.

Second, the training cost $C_{\text{train}}$ in Table 4 accounts strictly for the temporal advancements of the DRL training episodes ($t_{\text{train}}^{+}$) and excludes the one-time computational cost of generating the target reference flow state ($C_{\text{ref}}$). In canonical turbulent flow studies targeting standard regimes (e.g., turbulent channel flows at $Re_{\tau }=180,590,2000$), researchers routinely rely on pre-existing, publicly available DNS benchmark databases (Hoyas and Jiménez, 2006, Lee and Moser, 2015, Moser et al., 1999), which introduce zero additional computational cost to the flow control training workflow. In this work, because a ready-to-use public DNS benchmark case for the channel flow at $Re_{\tau }=100$ was not readily available, a dedicated uncontrolled baseline DNS was executed. As detailed in Section 4.2, this reference state for Case I ($Re_{\tau }=100$) is obtained by time-averaging the uncontrolled baseline DNS over 400FTTs. However, this custom baseline simulation constitutes a foundational, one-time research infrastructure investment rather than a recurring cost of policy training. Just as public DNS repositories serve as persistent benchmark assets for the broader fluid dynamics community, once this highly converged reference case is generated, it is retained and reused across multiple control policy implementations, state-reward formulations, network architecture evaluations, and hyperparameter tuning studies. Consequently, charging the full computational cost of building this reusable, multi-run reference database exclusively to a single trained policy instance fundamentally misrepresents the true marginal computational cost of policy training itself. Nevertheless, to provide complete transparency, generating this 400FTTs reference profile ($t_{{\text{avg}}_{\text{C}}}=3.47\cdot 10^{2}\;\text{s}$ or $t_{{\text{avg}}_{\text{C}}}^{+}=3.47\cdot 10^{4}$; see Section 4.2 for detailed setup) requires $N_{\text{t,ref,c}}=3.47\cdot 10^{6}$ conservative integration steps ($\Delta t_{c}^{+}$) or $N_{\text{t,ref,op}}=6.43\cdot 10^{4}$ CFL-optimal steps ($\Delta t_{\text{op}}^{+}$). Therefore, if 100% of $C_{\text{ref}}$ is conservatively charged to a single trained policy instance ($C_{\text{train,total}}=C_{\text{train}}+C_{\text{ref}}$), the resulting amortization thresholds shift to M≥43 (exploratory training) and M≥36 (early-stopped training) under conservative time-stepping, and to M≥21 (exploratory training) and M≥18 (early-stopped training) under CFL-optimal time-stepping, demonstrating that the framework maintains computational viability over repeated deployment runs even under this worst-case scenario.

Third, this cost analysis assumes that the total integration time required to satisfy the fluctuating velocity error tolerances ($\epsilon_{1}$, $\epsilon_{2}$, and $\epsilon_{3}$) across the deployment range ($Re_{\tau }\in \left[100,180\right]$) is upper-bounded by the temporal window required to achieve the targeted error tolerances for the most demanding evaluation setup (Case II, $Re_{\tau }=180$).

Fourth, the training, deployment, and baseline computational costs C are modeled as directly proportional to the total number of advanced temporal integration steps, $N_{\text{t}}$, and independent of $Re_{\tau }$. This formulation introduces the idealized assumption that the computational overhead relative to spatial discretization remains constant across the considered fluid domain boundaries ($Re_{\tau }\in \left[100,180\right]$). This implies ideal parallel scalability, where unconstrained hardware resources scales alongside the spatial grid resolution with no performance penalties. Crucially, this assumption results in a conservative calculation of the amortization threshold $M_{\text{min}}$. In real-world high-performance computing environments, the true behavior of computational cost scales as $C\propto N_{\text{dof}}N_{\text{t}}$, where $N_{\text{dof}}$ is the number of spatial degrees of freedom. Because the training phase is executed entirely at a low grid resolution (see Table 1 for Case I at $Re_{\tau }=100$), while the deployment and baseline evaluations are performed at a significantly higher grid resolution (see Table 1 for Case II at $Re_{\tau }=180$), the real spatial grid-dependent computational cost of the evaluation runs increases substantially relative to the lower fixed investment of the training phase. This scaling behavior dramatically amplifies the absolute computational savings achieved per deployment run ($C_{\text{base}}-C_{\text{deploy}}$) relative to $C_{\text{train}}$. Consequently, accounting for true spatial grid scaling would yield significantly lower values of $M_{\text{min}}$, demonstrating an even faster amortization of the framework’s computational cost during deployment.

Fifth, this computational cost and amortization analysis assumes that the learned REP-DRL policy generalizes to the target deployment regime without requiring full retraining. Conversely, if the flow condition departs substantially from the training regime such that policy transfer degrades, deploying the framework in a new regime requires retraining a new agent ($C_{\text{train,new}}$) and potentially generating a new target reference ($C_{\text{ref,new}}$). Under such cross-regime retraining, the framework cannot amortize its cost on a single deployment run (M=1). Instead, net computational savings are obtained only if the newly trained agent is deployed $M>M_{\text{min,new}}$ times within that new flow regime, a standard practice in computational fluid dynamics workflows such as ensemble DNS runs, uncertainty quantification, or parametric studies. This operational constraint highlights the core computational advantage of the cross-Reynolds-number transfer demonstrated in this work: training once in an inexpensive low-Reynolds-number regime and deploying in higher-Reynolds-number regimes without requiring additional retraining or reference generation costs.

Sixth, extending the framework to complex flow regimes using the full 6-D action space introduces a critical trade-off between PPO algorithm complexity and expanded physical control capabilities that directly impacts computational amortization. On one hand, exploring a higher-dimensional action space increases sample complexity, which is expected to moderately elevate the training cost ($C_{\text{train}}$) due to the additional exploratory episodes required. On the other hand, the reduced 3-D action space lacks the rotational freedom to manipulate the $R_{ij}$ principal axes in generic non-homogeneous flows, resulting in diminished convergence acceleration ($C_{\text{deploy}}\approx C_{\text{base}}$). In contrast, as introduced in Section 5.1, the full 6-D action space is expected to achieve greater statistics convergence acceleration during deployment ($C_{\text{deploy}}\ll C_{\text{base}}$) in non-homogeneous flows, ultimately lowering the amortization threshold ($M_{\text{min}}$) and reinforcing the practical viability of the REP-DRL framework for engineering applications.

## 6 Conclusions

This work introduced and explored the potential of a novel Reynolds stresses eigenspace perturbation (REP)-based deep reinforcement learning framework, termed REP-DRL, as a proof-of-concept for accelerating the statistical convergence of direct numerical simulations of turbulent flow. This approach utilizes a multi-agent proximal policy optimization controller to learn an optimal control policy that applies a physically-derived, volumetric forcing term based on local Reynolds stresses perturbations. This framework is trained in a supervised manner on a $Re_{\tau }=100$ turbulent flow (Case I), and subsequently evaluated in an unsupervised setting on both the training case and a higher-Reynolds number turbulent flow (Case II, $Re_{\tau }=180$) to assess its performance and explore its generalization capabilities.

The exploration of this framework demonstrated significant and promising results. The supervised training process provided valuable insights into the control problem, including the identification of a performance trade-off, necessitating the selection of a compromise policy. This trained policy successfully accelerated the convergence of the velocity fluctuations and the underlying turbulent structure (anisotropy) in the unsupervised evaluation of the $Re_{\tau }=100$ case. The generalization test on $Re_{\tau }=180$ case also provided a key finding: while long-term integration proved unstable for this preliminary REP-DRL implementation, it still provided an initial convergence acceleration of the velocity fluctuations. Moreover, the comparison between the unsupervised evaluations at $Re_{\tau }=100$ and $Re_{\tau }=180$ reveals increasing long-term deviations as the flow departs from the training regime, particularly in the mean streamwise velocity. This behavior indicates a limited transferability of the current policy across Reynolds number variations and sufficiently distinct turbulent flow regimes, suggesting that robust generalization would require training on a curriculum of flow conditions. Furthermore, the sensitivity analysis on the actuation period further reinforced this potential, revealing that different control frequencies could be optimized to target specific statistical moments. This indicates that the observed performance trade-offs are a fundamental aspect of the control problem, highlighting a clear research path toward more advanced, multi-objective optimization strategies. Finally, a rigorous end-to-end computational cost analysis confirms the practical viability of this approach for high-fidelity turbulence simulations. The initial $Re_{\tau }=100$ training overhead is rapidly amortized during deployment at $Re_{\tau }=180$. Depending on the training duration and early-stopping criteria, the computational investment under the executed conservative time steps is recovered within 3 to 10 independent runs, where the 3 runs threshold corresponds to retrospective early stopping at episode 17. Under a projected CFL-optimal time step, this amortization threshold decreases to 2 to 5 deployment runs, with 2 runs representing an idealized lower bound that combines retrospective early stopping with numerical time step optimization. Because this amortization cost study relies on a single training trajectory and policy checkpoint, these resulting amortization thresholds should be interpreted as a proof-of-concept baseline. Nevertheless, this assessment relies on intentionally conservative benchmarks of constant spatial discretization cost; real-world high performance computing configurations, where costs scale with spatial degrees of freedom, would yield even faster amortization. However, this computational cost amortization is subject to operating within a comparable Reynolds number regime, whereas retraining required for operating at substantially different flow conditions would increase the effective training cost and thereby reduce the amortization advantage. These findings establish the REP-DRL framework as a promising approach for bypassing the severe temporal integration bottlenecks associated with converging high-order turbulence statistics such as fluctuating velocity components.

Building on these findings, future work will focus on improving policy robustness and generalization. In this context, a necessary first step involves a systematic assessment of training robustness with respect to independent training seeds, stochastic policy initialization, and distinct restart flow fields, which is currently beyond the scope of this proof-of-concept study. Specifically, multi-seed training ensembles would enable the quantification of across-seed variance and the establishment of confidence intervals for deployment metrics and computational amortization. Robustness requirements can also be addressed by training the control agents on progressively more complex flow conditions, such as varying Reynolds numbers (Zhou et al., 2025). Furthermore, to address the identified performance trade-offs and limitations of the current scalarized reward function, a true multi-objective reinforcement learning (MORL) approach (Roijers et al., 2013, Yang et al., 2019) could be explored to learn the Pareto front of policies, explicitly managing the balance between converging different statistics. The state representation could also be enhanced by incorporating graph neural networks (GNNs) to share information between neighboring agents (Kurz et al., 2025), or by introducing history-dependent formulations such as recurrent neural networks (e.g., long short-term memory (LSTM) (Hausknecht & Stone, 2017)) or history-augmented states. These approaches may help mitigate partial observability effects and thereby improve policy robustness and generalization. Additionally, the high cost of direct numerical simulations motivates exploring off-policy algorithms, such as soft actor-critic (SAC) (Haarnoja et al., 2018), which could significantly improve sample efficiency. Finally, to address the critical instabilities observed during generalization, physics-informed reinforcement learning (PIRL) techniques could be integrated (Banerjee et al., 2025) to ensure safe exploration. Beyond these algorithmic and training enhancements, future studies will extend the simulation horizons past the active control window to systematically map the post-actuation continuation dynamics under unforced conditions ($f_{\text{REP}}=0$). Because running time-averages collected under active forcing sample a forced phase-space distribution, post-actuation continuation simulations are required to quantitatively characterize how the instantaneous flow dynamics and cumulative statistics relax from the perturbed state back toward the unforced dynamics. Finally, future research should focus on scaling the implementation to the full six-dimensional action space to perform $R_{ij}$ principal axes rotation, enabling the framework to modify shear-stress generation and dynamically accommodate stress–strain misalignments in complex flow configurations (Gorlé and Iaccarino, 2013, Thompson et al., 2019).

## CRediT authorship contribution statement

**Núria Masclans:** Conceptualization, Formal analysis, Investigation, Software, Writing – original draft. **Lluís Jofre:** Conceptualization, Funding acquisition, Investigation, Supervision, Writing – review & editing.

## Funding sources

This work is funded by the European Union (ERC, SCRAMBLE, 101040379). Views and opinions expressed are however those of the authors only and do not necessarily reflect those of the European Union or the European Research Council. Neither the European Union nor the granting authority can be held responsible for them.

## Declaration of competing interest

The authors declare that they have no known competing financial interests or personal relationships that could have appeared to influence the work reported in this paper.

## Figures and Tables

**(a):**
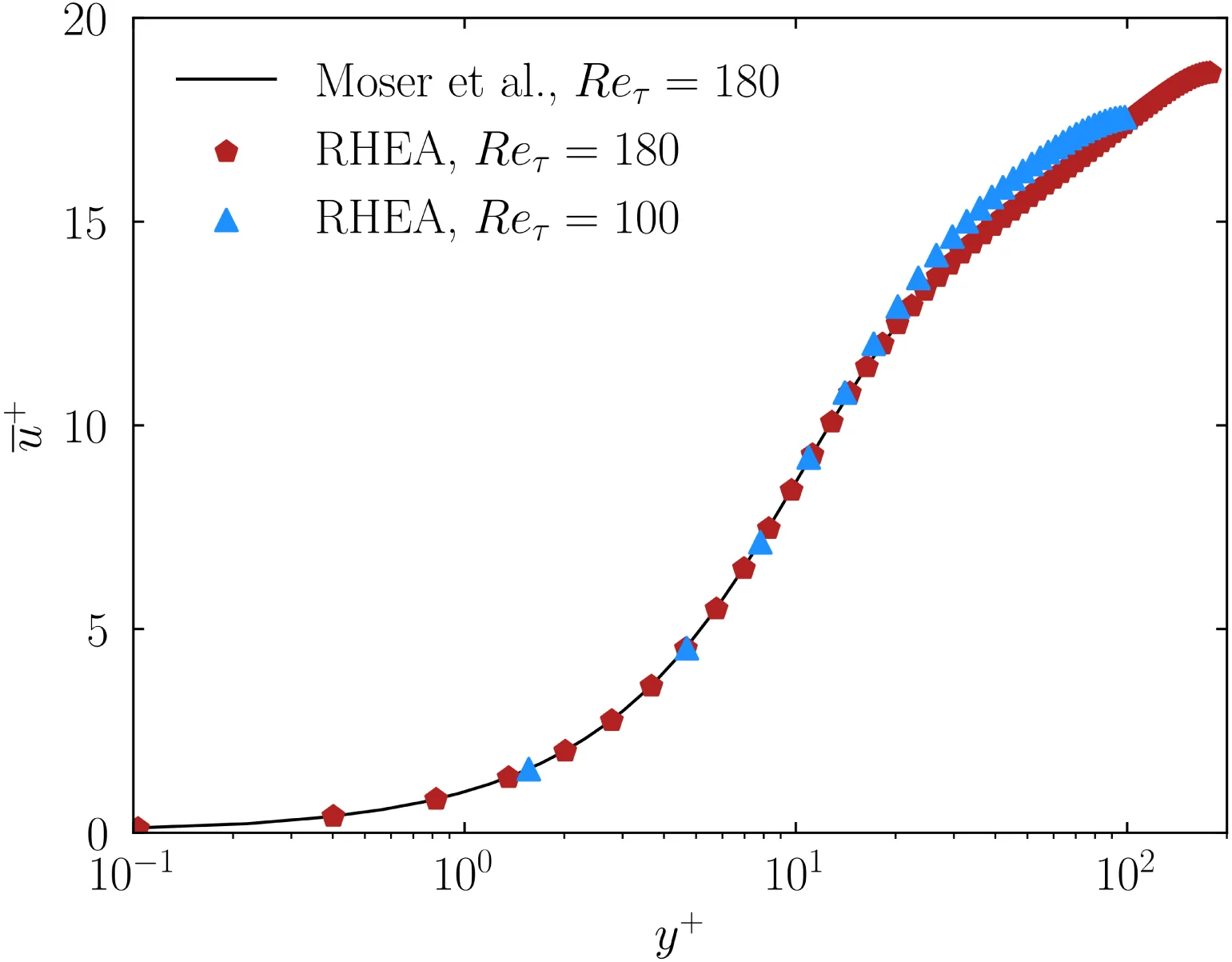
.

**(b):**
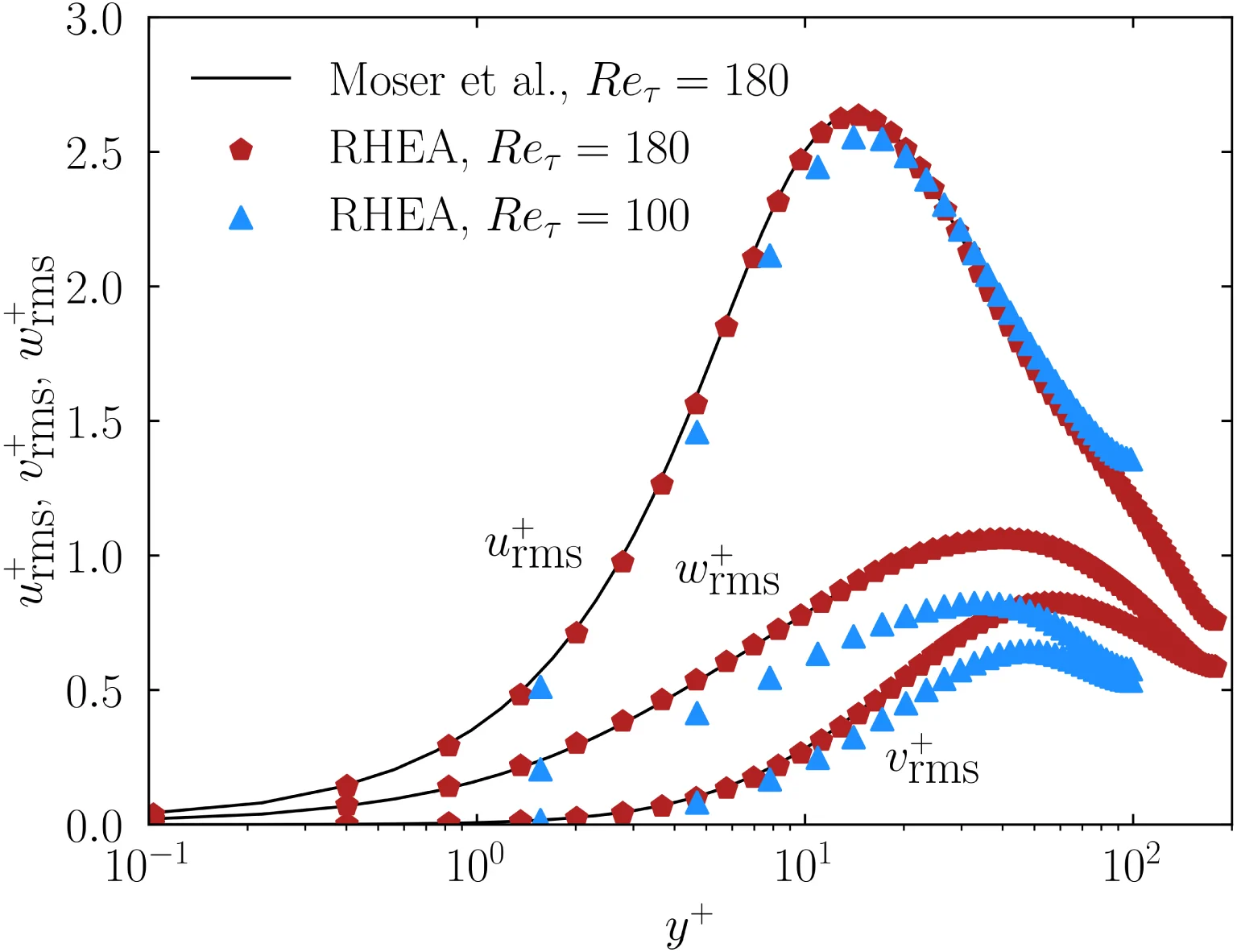
.

## Data Availability

Data will be made available on request.

## Appendix A Turbulent channel flow validation

This appendix presents the validation of the flow physics modeling in the RHEA flow solver (Abdellatif et al., 2025, Jofre et al., 2023, Monteiro et al., 2026) for the benchmark problem of 3-D turbulent channel flow. The solver’s accuracy is assessed by comparing converged first- and second-order velocity statistics from RHEA against the well-established DNS results of Moser et al. (1999). Validation is conducted at a friction Reynolds number of $Re_{\tau }=180$, for which this reference data is available. This validation provides confidence in the solver’s accuracy for the $Re_{\tau }=100$ case, which lacks a reference dataset.

The computational setup for $Re_{\tau }=180$ follows the methodology described by Jofre et al. (2023), while the setup for $Re_{\tau }=100$ is detailed in Section 4.2. In both cases, statistical averaging is initiated after an initial transient period of 50 FTTs, and the reference statistics are then averaged for approximately 400 FTTs for $Re_{\tau }=100$ and 173 FTTs for $Re_{\tau }=180$ to ensure full statistical convergence.

Fig. A.12 compares the (a) mean streamwise velocity (${\bar{u}}^{+}$) and (b) root-mean-square (rms) velocity fluctuations ($u_{\text{rms}}^{+}$, $v_{\text{rms}}^{+}$ and $w_{\text{rms}}^{+}$) along the wall-normal direction in wall units. The ${\bar{u}}^{+}$ profiles presented in Fig. A.12(a) show excellent agreement between RHEA and the reference data at $Re_{\tau }=180$ across the entire domain. For $Re_{\tau }=100$, the RHEA ${\bar{u}}^{+}$ profile follows the expected physical trend, with slightly lower ${\bar{u}}^{+}$ values in the inner region due to the reduced Reynolds number. The fluctuating velocity profiles depicted in Fig. A.12(b) further validate the solver’s capabilities. The RHEA fluctuation profiles align well with the reference data at $Re_{\tau }=180$, while the $Re_{\tau }=100$ case exhibits lower overall turbulence intensity, consistent with known Reynolds number effects. These results validate the ability of the RHEA flow solver to accurately capture the relevant physics of wall-bounded turbulence and provide confidence on its application to the $Re_{\tau }=100$ and $Re_{\tau }=180$ turbulent channel flow cases utilized in this work.

## Appendix B Policy gradient methods

Policy gradient (PG) methods are a class of RL algorithms that directly learn a parameterized policy, $\pi_{\theta }\left(a|s\right)$, by performing gradient ascent on the expected cumulative reward. The foundation of this approach was established by the REINFORCE algorithm (Williams, 1992), which is one of the earliest PG methods that updates policy parameters via gradient ascent on the expected return. The algorithm’s objective is to find the optimal policy parameters θ that maximize the expected return J(θ). Formally, the return R(τ) for a given trajectory τ is the cumulative sum of discounted rewards in the form

(B.1) $$R\left(\tau \right)=\overset{T}{\underset{t=0}{\sum }}\gamma^{t}r_{t},$$

where γ∈[0,1] is the discount factor and $r_{t}=r\left(s_{t}\right)$ is the reward at time step t. The objective function J(τ) is the expected return over all trajectories sampled from the policy $\pi_{\theta }$ as

(B.2) $$J\left(\theta \right)=E_{\tau ∼\pi_{\theta }}\left[R\left(\tau \right)\right],$$

and the optimization goal is to find the policy parameters $\theta^{⋆}$ that maximize this objective given by

(B.3) $$\theta^{⋆}=\arg\underset{\theta }{\max}J\left(\theta \right).$$

This optimization problem is solved by iteratively updating the policy parameters θ via gradient ascent as

(B.4) $$\theta_{k+1}\leftarrow \theta_{k}+\alpha \nabla_{\theta }J\left(\theta_{k}\right),$$

where α is the learning rate and $\nabla_{\theta }J\left(\theta_{k}\right)$ represents the policy gradient at the kth policy update iteration. The PG theorem provides an expression for the policy gradient $g=\nabla_{\theta }J\left(\theta \right)$ in terms of the sampled trajectories as

(B.5) $$g=E_{\tau ∼\pi_{\theta }}\left[\nabla_{\theta }\log\pi_{\theta }\left(a_{t}|t_{s}\right)\;R_{t}\left(\tau \right)\right],$$

which is commonly computed using REINFORCE gradient estimator (Williams, 1992) in the form

(B.6) $$\overset{ˆ}{g}={\overset{ˆ}{E}}_{t}\left[\nabla_{\theta }\log\pi_{\theta }\;{\overset{ˆ}{G}}_{t}\right],$$

where ${\overset{ˆ}{E}}_{t}$ is the empirical average over a finite batch of samples, and ${\overset{ˆ}{G}}_{t}=\sum_{k=t}^{T}\gamma^{k-t}r_{k}$ is the empirical return from time t, often referred to as the reward-to-go. However, this gradient estimator exhibits high variance, which can be significantly reduced by introducing a state-dependent baseline, such as the value function V(s), to improve convergence (Sutton et al., 1999). This leads to the use of the advantage function $A_{t}=A^{\pi }\left(s_{t},a_{t}\right)$, which quantifies whether an action is better or worse than the policy’s averaged behavior at that state. The resulting policy gradient estimator $\overset{ˆ}{g}$ has lower variance while maintaining the same expected value, yielding

(B.7) $$\overset{ˆ}{g}={\overset{ˆ}{E}}_{t}\left[\nabla_{\theta }\log\pi_{\theta }\left(a_{t}|t_{s}\right){\overset{ˆ}{A}}_{t}\right].$$

This policy gradient is the gradient of the surrogate objective function

(B.8) $$L^{\text{PG}}\left(\theta \right)={\overset{ˆ}{E}}_{t}\left[\log\pi_{\theta }\left(a_{t}|t_{s}\right)\;{\overset{ˆ}{A}}_{t}\right],$$

which is what PG methods aim to maximize. PG methods optimize the policy parameters θ by performing gradient ascent on this objective, using the policy gradient estimator $\overset{ˆ}{g}$ from (B.7) in each update step.

In practice, standard deep learning libraries are configured for loss function minimization. To align with this architecture, the policy gradient maximization objective $L^{PG}\left(\theta \right)$ is implemented by defining the loss function as its negative, $L\left(\theta \right)=-L^{\text{PG}}\left(\theta \right)$, which is then minimized using a standard gradient descent optimizer. This formulation serves as the foundation for modern PG algorithms, including actor-critic methods such as TRPO (Schulman, Levine, et al., 2015) and PPO (Schulman et al., 2017), detailed in Appendix C.

## Appendix C Proximal policy optimization

Proximal policy optimization (PPO) is an on-policy, actor-critic algorithm that builds upon the PG method described in Appendix B (Schulman et al., 2017). Its primary innovation is to constrain the policy updates to prevent the destructively large, unstable changes that often lead to performance collapse. This is achieved by optimizing a surrogate objective function, originally derived in the context of conservative policy iteration (CPI) (Kakade & Langford, 2002)

(C.1) $$L^{\text{CPI}}\left(\theta \right)={\overset{ˆ}{E}}_{t}\left[r_{t}\left(\theta \right){\overset{ˆ}{A}}_{t}\right],$$

where ${\overset{ˆ}{A}}_{t}={\overset{ˆ}{A}}^{\pi_{\text{old}}}\left(s_{t},a_{t}\right)$ is the advantage function estimator computed using the previous policy $\pi_{\text{old}}$, and $r_{t}\left(\theta \right)$ is the probability ratio between the new policy, $\pi_{\theta }$ and the previous policy, $\pi_{\theta_{\text{old}}}$, calculated as

(C.2) $$r_{t}\left(\theta \right)=\frac{\pi_{\theta }\left(a_{t}|t_{s}\right)}{\pi_{\theta_{\text{old}}}\left(a_{t}|t_{s}\right)}.$$

Directly maximizing $L^{\text{CPI}}\left(\theta \right)$ with multiple gradient steps is notoriously unstable, as $r_{t}\left(\theta \right)$ can become arbitrarily large. To mitigate this instability that leads to excessively large policy updates, PPO introduces a trust-region constraint into the optimization problem. One approach to enforcing this constraint is to explicitly penalize the Kullback–Leibler (KL) divergence between the new and old policies, leading to the KL-penalized surrogate objective to maximize

(C.3) $$L^{\text{KLPEN}}\left(\theta \right)={\overset{ˆ}{E}}_{t}\left[r_{t}\left(\theta \right){\overset{ˆ}{A}}_{t}-\beta \;\text{KL}\left(\pi_{\theta }\left(a_{t}|t_{s}\right)∥\pi_{\theta_{\text{old}}}\left(a_{t}|t_{s}\right)\right)\right],$$

where β is the KL penalty coefficient that controls the strength of the KL regularization.

An alternative approach is the clipped surrogate objective, which avoids explicit KL constraints by restricting the probability ratio updates within a predefined range. The clipped surrogate objective is formulated as

(C.4) $$L^{\text{CLIP}}\left(\theta \right)={\overset{ˆ}{E}}_{t}\left[\min\left(r_{t}\left(\theta \right){\overset{ˆ}{A}}_{t},\;\text{clip}\left(r_{t}\left(\theta \right),1-\epsilon ,1+\epsilon \right){\overset{ˆ}{A}}_{t}\right)\right],$$

where ϵ is the clipping threshold that defines the trust region for policy updates. The clipped PPO is adopted in the present implementation due to its computational efficiency, implementation simplicity, and robust performance compared to KL-penalized PPO (Schulman et al., 2017).

As an actor-critic method, PPO also trains a critic network, $V_{\phi }\left(s\right)$, which represents the state-value function and is essential for computing a variance-reduced advantage estimator ${\overset{ˆ}{A}}_{t}$, as exposed in Appendix B. This critic network is optimized simultaneously with the actor by minimizing a standard squared-error loss, $L_{\text{VF}}$, via gradient descent as

(C.5) $$L^{\text{VF}}\left(\phi \right)={\overset{ˆ}{E}}_{t}\left[{\left(V_{\phi }\left(s_{t}\right)-V_{t}^{\text{targ}}\right)}^{2}\right],$$

where the value target $V_{t}^{\text{targ}}$ is an empirical estimate of the expected return. This target is typically defined as the reward-to-go ${\overset{ˆ}{G}}_{t}$, defined in Appendix B, or a more advanced, lower-variance estimate such as that provided by the generalized advantage estimation (GAE) (Schulman, Moritz, et al., 2015b) or the finite-horizon estimator (Mnih et al., 2016).

Therefore, the final PPO objective to maximize is a composite function that combines the actor’s clipped objective (C.4), the critic’s value loss (C.5), and an entropy bonus term S to encourage exploration in the form (Schulman et al., 2017)

(C.6) $$L_{t}\left(\theta ,\phi \right)={\overset{ˆ}{E}}_{t}\left[L_{t}^{\text{CLIP}}\left(\theta \right)-\beta_{1}L_{t}^{\text{VF}}\left(\phi \right)+\beta_{2}S\left[\pi_{\theta }\right]\left(s_{t}\right)\right],$$

where $\beta_{1}$ and $\beta_{2}$ are weighting coefficients, and $S\left[\pi_{\theta }\right]\left(s_{t}\right)$ is the entropy of the policy at state $s_{t}$. Here, the value loss term $L_{t}^{\text{VF}}$ is subtracted, as it represents a loss to be minimized, whereas the policy objective $L_{t}^{\text{CLIP}}$ and the entropy bonus S are terms to be maximized.

## Appendix D Open source codes

The source code for the presented DRL-CFD implementation for statistical convergence acceleration is released as open source on a GitHub repository (Masclans, 2026). The CFD simulation environment is based on RHEA (Abdellatif et al., 2025, Jofre et al., 2023, Monteiro et al., 2026), an open-source reproducible hybrid-architecture flow solver engineered for academia, while the DRL framework is implemented using TF-Agents (Guadarrama et al., 2018), a TensorFlow library for RL applications. To facilitate seamless communication between the Python-based DRL model and the parallelized C++ solver, the SmartSim (Partee et al., 2022) library is employed. SmartSim enables in-memory communication through a Redis database, achieving low-latency, memory-efficient exchange of states, actions, and rewards while minimizing software complexity.
